# Aptamer-Based Biosensors for the Detection of Malaria

**DOI:** 10.3390/bios16090456

**Published:** 2026-08-23

**Authors:** Josep J. Centelles, Santiago Imperial

**Affiliations:** Department of Biochemistry and Molecular Biomedicine, School of Biology, University of Barcelona, Av. Diagonal 643, 08028 Barcelona, Spain; josepcentelles@ub.edu

**Keywords:** malaria, aptamer, aptasensor, malaria biomarkers, *Plasmodium*

## Abstract

Malaria remains one of the most significant infectious diseases worldwide, requiring rapid, sensitive, and accessible diagnostic tools to improve disease management and control. Conventional diagnostic methods, including microscopy, rapid diagnostic tests, and nucleic acid amplification techniques, present limitations in sensitivity, specificity, cost, or field applicability. This review examines the emerging role of aptamer-based biosensors (aptasensors) as innovative alternatives for malaria detection. Aptamers are synthetic nucleic acid ligands that offer high affinity and specificity toward malaria biomarkers while providing advantages over antibodies, including improved stability, lower production costs, and ease of chemical modification. The review discusses aptamer selection methodologies, major *Plasmodium* biomarkers targeted for detection, and the integration of aptamers into electrochemical, optical, magnetic, and microfluidic biosensing platforms. Current advances demonstrate the potential of aptasensors to enable highly sensitive, selective, and portable point-of-care diagnostics for malaria surveillance and management.

## 1. Malaria Description and Incidence

Malaria is an acute, febrile illness caused by protozoan parasites of the genus *Plasmodium*, transmitted to humans primarily through the bite of infected female *Anopheles* mosquitoes [1,2]. This illness remains a major global health crisis, particularly in tropical and subtropical developing nations [3,4]. In its latest report, published in December 2025, the World Health Organization (WHO) estimated 282 million malaria cases and reported 610,000 malaria-related deaths worldwide for 2024 [5]. Nearly half of the world’s population is currently at risk of malaria [5,6,7].

Five species of *Plasmodium* routinely infect humans: *P. falciparum*, *P. vivax*, *P. ovale*, *P. malariae*, and *P. knowlesi*, with *P. falciparum* causing the most severe and fatal form of the disease [7,8,9,10,11].

**Figure 1:**
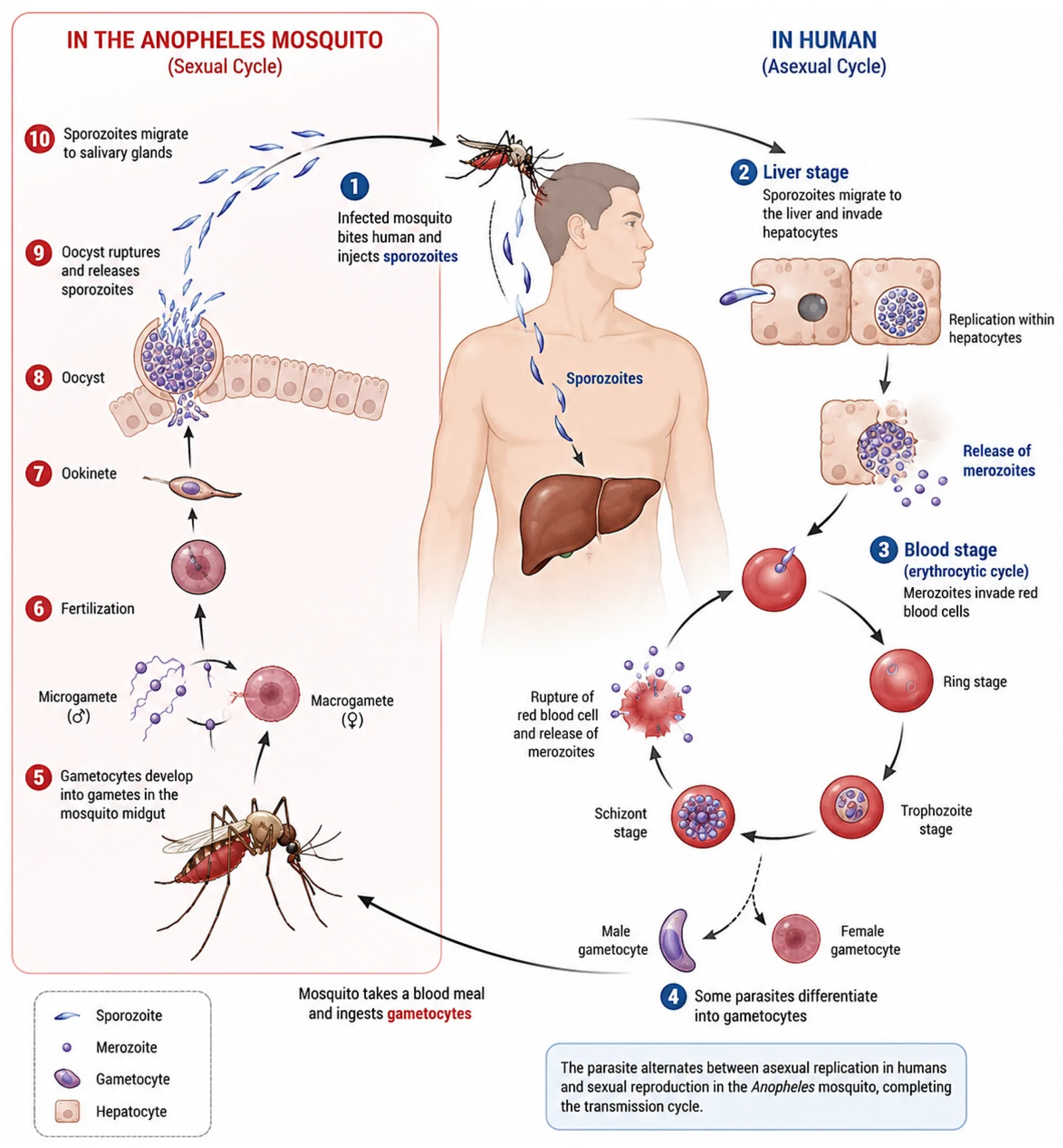
Life cycle of *Plasmodium* spp. in humans and the *Anopheles* mosquito. Following the bite of an infected female *Anopheles* mosquito, sporozoites enter the human bloodstream and migrate to the liver, where they undergo replication and differentiation into merozoites. Merozoites are subsequently released into the bloodstream and invade red blood cells, progressing through the ring, trophozoite, and schizont stages of the asexual erythrocytic cycle responsible for the clinical manifestations of malaria. Some parasites differentiate into gametocytes, which are ingested by mosquitoes during a blood meal, where sexual reproduction occurs, completing the transmission cycle. The illustration was created using generative AI tools.

The disease follows a complex life cycle involving an asexual erythrocytic stage in the human host that is responsible for clinical symptoms. Sporozoites, introduced by the mosquito, travel to the liver, replicate, and differentiate into merozoites [12]. Merozoites are then released into the bloodstream to invade red blood cells (RBCs), leading to the symptomatic asexual cycle that progresses through ring, trophozoite, and schizont stages [12]. See Figure 1 for a more detailed life cycle of *Plasmodium*.

*P. vivax* and *P. ovale* can form dormant liver-stage parasites known as hypnozoites that can persist for months or years, causing disease relapses [6,13,14]. Eliminating *P. vivax* requires a ‘radical cure’, typically involving 8-aminoquinolines like primaquine or tafenoquine, which target these liver stages [14,15].

The clinical manifestations of severe malaria are linked to several biochemical and physiological perturbations in the host: During the intraerythrocytic stage, *Plasmodium* parasites consume host hemoglobin as an amino acid source, releasing highly toxic free heme [16,17,18,19,20].

To detoxify this heme, the parasite biomineralizes it into hemozoin, an inert, insoluble, paramagnetic biocrystal localized in the parasite’s digestive vacuole [16]. Hemozoin presence is a direct indicator of metabolically active parasites [13,21].

Malaria infection frequently disrupts glucose metabolism, leading to hypoglycemia, primarily resulting from parasite consumption of circulating glucose, sequestration of infected RBCs in the liver, and altered insulin response [6]. Dyslipidemia is also commonly observed, characterized by elevated triacylglycerol (TAG) and free fatty acid levels, alongside decreased HDL, LDL, and total cholesterol, as the parasite manipulates host lipid levels for its own growth and development [6].

Liver dysfunction, indicated by sharp rises in aspartate transaminase and alanine transaminase, results from congestion, cellular inflammation, and sinusoidal blockage [6]. Kidney damage may also occur, leading to complications like acute renal failure [6]. Furthermore, the host immune response and parasite metabolic processes generate reactive oxygen species and reactive nitrogen species in excess of the host’s total antioxidant capacity, causing oxidative stress that contributes to endothelial damage and complicated malaria [6].

## 2. Malaria Detection Methods

Accurate and timely diagnosis is essential for effective case management, surveillance, and treatment intervention [13]. Malaria diagnosis techniques can be classified into conventional detection methods and detection methods using biosensors.

### 2.1. Conventional Detection Methods

Light microscopy (LM), specifically examining Giemsa-stained thick and thin blood films, is considered the gold standard for clinical malaria diagnosis [6,16]. It allows for species and stage identification and parasitemia quantification, offering an excellent view of the parasite [13]. However, LM is resource-intensive, time-consuming, and depends heavily on the microscopist’s skill [6,16]. Its typical lower limit of detection ranges from 50 to 200 parasites/µL of blood under field conditions [16,22,23].

Rapid Diagnostic Tests (RDTs) are lateral flow immunoassays integrated into cassettes that are popular for point-of-care (POC) applications due to their simplicity, speed, and portability [6,23,24,25,26,27,28]. Most malaria RDTs currently manufactured and deployed rely on the use of antibodies to detect *Plasmodium falciparum* histidine-rich protein 2 (PfHRP2), either as a standalone biomarker or in combination with other parasite antigens such as lactate dehydrogenase (LDH) and aldolase. Despite their widespread use, antibody-based diagnostic platforms present several limitations [29]. These include a relatively short shelf life, typically around nine months, reduced stability under the high-temperature and high-humidity conditions frequently encountered in endemic regions, and dependence on animal-derived or hybridoma-produced antibodies, which are associated with elevated production costs and potential batch-to-batch variability. Furthermore, the intracellular lifestyle of *Plasmodium* parasites restricts the number of validated diagnostic targets available, and some of these targets may be absent in specific parasite populations, as demonstrated by the emergence of *pfhrp2*-deleted strains that compromise the performance of PfHRP2-based RDTs [30,31]. In addition, most commercially available RDTs have been optimized for the detection of *P. falciparum*, whereas assays targeting non-*falciparum* species generally exhibit lower diagnostic sensitivity [32]. Beyond primary diagnosis, antigen detection assays are also used to assess treatment efficacy and parasite clearance; however, circulating parasite antigens may persist in the bloodstream after parasitemia has been eliminated, resulting in false-positive test outcomes [33]. Consequently, the development of more accurate, robust, and reliable diagnostic approaches remains a critical objective in malaria control and management.

The sensitivity of RDTs typically stands at around 100–200 parasites/µL of blood, often failing to detect low-level parasitemia [4,13,16].

Nucleic Acid Amplification Test techniques like Polymerase Chain Reaction (PCR) and Loop-Mediated Isothermal Amplification (LAMP) offer superior sensitivity, capable of detecting levels as low as 5 parasites/µL [6,13,16,34]. While PCR is the gold standard in centralized laboratories, it requires complex thermal cycling [34]. LAMP is preferred for POC settings because it is isothermal, faster, and more robust to inhibitors, making the instrumentation less complex and less costly [34].

### 2.2. Detection Methods Using Biosensors

Biosensors are integrated analytical devices composed of an immobilized biorecognition element (e.g., antibody, aptamer, enzyme) coupled to a transducer that converts a binding event into a measurable signal [6,16,35,36,37]. An ideal POC device is often summarized by the WHO criteria as ASSURED, i.e., Affordable, Sensitive, Specific, User-friendly, Rapid and Robust, Equipment-free, and Deliverable [13,38]. Biosensors aim to close the diagnostic gap left by LM and RDTs by offering high sensitivity, portability, and throughput [13,16].

Biosensor-based detection methods have emerged as a powerful approach to address the limitations of conventional malaria diagnostics, offering enhanced sensitivity, rapid response times, and suitability for point-of-care applications. Biosensors target known protein biomarkers (PfHRP-2, pLDH, PfGDH) [13] but also unique parasite-specific indicators like hemozoin [16,21]. Central to the performance of biosensors is the choice of the biorecognition element, and in this context, aptamers have gained increasing attention as highly effective molecular probes for malaria detection [39,40,41].

## 3. Aptamers: Properties, Selection Methodologies, and Advantages

The following section introduces aptamers, including how they are prepared and selected, as well as their main advantages compared with traditional antibody-based methods. Aptamers are short single-stranded nucleic acids capable of binding specific targets with high affinity and specificity, making them promising alternatives to antibodies in diagnostics, therapeutics, and biosensing applications. Their chemical stability, lower production costs, and ease of modification provide several benefits over conventional antibody technologies.

### 3.1. Aptamers

Aptamers are synthetic single-stranded nucleic acid molecules (DNA or RNA) that usually range from 20 to 100 bases in length [35,36,42]. These molecules are often referred to as “chemical antibodies” because they are designed to fold into specific three-dimensional (3D) conformations (e.g., hairpins, loops, G-quadruplex structures) that bind to target molecules (ligands) with high affinity and specificity [35,36,38,43,44]. The binding strength is quantified by the dissociation constant, with some aptamers achieving picomolar affinity [42,43].

Aptamers present several advantages over traditional antibodies for biosensing applications [36,38,43]. These advantages include easier production, stability and reversibility, size and modification and target range. First, aptamers are produced entirely in vitro using chemical synthesis, eliminating the need for laboratory animals, saving time and costs, and ensuring high purity and low batch-to-batch variability [12,35,36,38,43]. Moreover, aptamers are remarkably stable and resistant to denaturation from harsh environments like extreme temperatures, pH, and humidity, which often degrade protein-based antibodies [12,36,38]. They can undergo reversible folding/unfolding, allowing for easy regeneration and reusability of the sensor surface [38]. Compared to antibodies, aptamers are significantly smaller (5–15 kDa vs. ~150 kDa) [12,36], facilitating high-density immobilization, superior penetration into tissues, and simple incorporation of functional groups (e.g., fluorophores, thiols) for immobilization or signal generation [37,43]. Finally, aptamers can be selected against targets that are non-immunogenic or toxic, including small molecules, metal ions, proteins, and even whole cells [36,37,43].

### 3.2. Methods of Aptamer Selection

**Figure 2:**
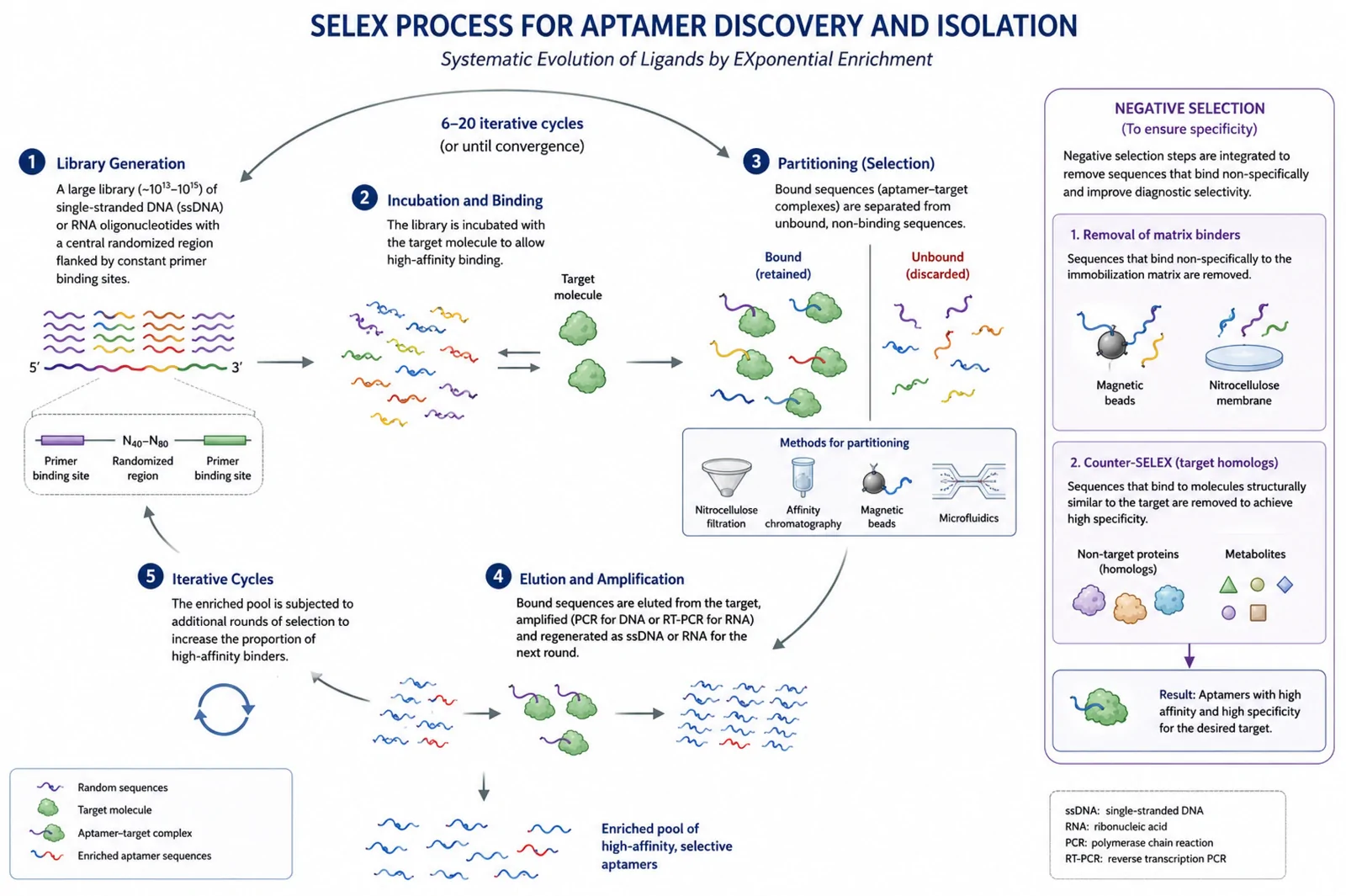
Schematic representation of the SELEX (Systematic Evolution of Ligands by EXponential Enrichment) process for aptamer selection. A randomized library of single-stranded DNA (ssDNA) or RNA oligonucleotides containing a central variable region flanked by constant primer binding sites is incubated with the target. Sequences that bind the target are separated from non-binding oligonucleotides through partitioning methods such as affinity chromatography, magnetic beads, nitrocellulose filtration, or microfluidic systems. Bound sequences are subsequently eluted, amplified by PCR (or RT-PCR for RNA aptamers), and regenerated for further selection rounds. Multiple iterative cycles of binding, partitioning, and amplification progressively enrich the pool in high-affinity aptamers. Negative selection and counter-SELEX steps are incorporated to eliminate sequences that bind non-specifically to the immobilization matrix or structurally related molecules, thereby improving target specificity and selectivity. The illustration was created using generative AI tools.

The primary method used to discover and isolate aptamers is the Systematic Evolution of Ligands by Exponential Enrichment (SELEX), an iterative in vitro selection and amplification procedure [35,38,42,43] (see Figure 2).

A description of the key steps in conventional SELEX follows:

Library Generation: Start with a large library of single-stranded DNA (ssDNA) or RNA oligonucleotides containing a central randomized region flanked by constant primer binding sites [12,38,42,43].

Incubation and Binding: The library is incubated with the target molecule under specific conditions to allow high-affinity binding [38,42,43].

Partitioning (Selection): Bound sequences (aptamer–target complexes) are separated from unbound, non-binding sequences. Early methods utilized nitrocellulose filters or affinity chromatography [43,45], while modern methods employ magnetic beads or microfluidics for faster and more stringent separation [38,45].

Elution and Amplification: Bound sequences are eluted from the target, amplified (via PCR for DNA, RT-PCR for RNA), and regenerated as ssDNA or RNA for the next round [38,42,43].

Iterative Cycles: This process is repeated 6–20 times until the pool is sufficiently enriched with high-affinity binders [42,43].

To ensure that the resulting aptamers are highly selective for the desired target and minimize non-specific binding, stringent negative selection steps are integrated into the process [38,43]. Sequences that bind non-specifically to the immobilization matrix (e.g., magnetic beads or nitrocellulose membranes) are removed [7,38]. Those sequences that bind to molecules structurally similar to the target (e.g., human homologous proteins or metabolites) are explicitly removed. This step is called Counter-SELEX and is crucial for achieving diagnostic selectivity [38,46,47,48].

Although SELEX was initially the most commonly used method to identify specific aptamers, different modifications have been developed; for instance, cell-SELEX uses whole live cells, infected tissues, or membranes as the target, enabling the discovery of aptamers targeting native cell-surface markers without prior knowledge of the antigens [45,49,50,51].

Inertial Microfluidic SELEX (I-SELEX) represents an innovation of the SELEX technique where centrifugal forces are utilized in spiral microfluidic channels to achieve highly efficient and continuous separation of micron-sized target cells (e.g., infected RBCs) from unbound aptamers in a single pass, enhancing selection stringency [49]. The technique achieved a high partitioning efficiency and successfully selected aptamers against *P. falciparum*-infected RBCs (IEs) [49].

Finally, other alternatives involve integrating chemically modified nucleotides (e.g., cubane-modified deoxyuridine triphosphate) into the aptamer library to confer improved stability, nuclease resistance, and novel binding chemistries not seen in natural nucleic acids [45,47].

## 4. Target Proteins for Aptamer Development in Malaria Detection

The following section introduces the rationale for the selection of the target proteins and describes the biomarkers commonly used for their identification and analysis. It also reviews the proteins and aptamers that have been identified and characterized so far, highlighting their relevance in diagnostic and therapeutic applications. Particular attention is given to the interaction between aptamers and target proteins, as these molecules offer high specificity and affinity for biomarker detection and molecular recognition.

Rather than a purely proteomic overview, this section focuses on the specific molecular handles that make *Plasmodium* proteins suitable for aptamer recognition. For instance, the unique five-residue insertion in pLDH is not merely a structural curiosity but the primary epitope used for species-specific discrimination in SPR and electrochemical sensors. Similarly, the repetitive motifs in PfHRP2 (e.g., “AHH”) are analysed here specifically for their ability to facilitate multivalent binding with G-quadruplex aptamers, which directly influences the sensor’s linear response range.

**Figure 3:**
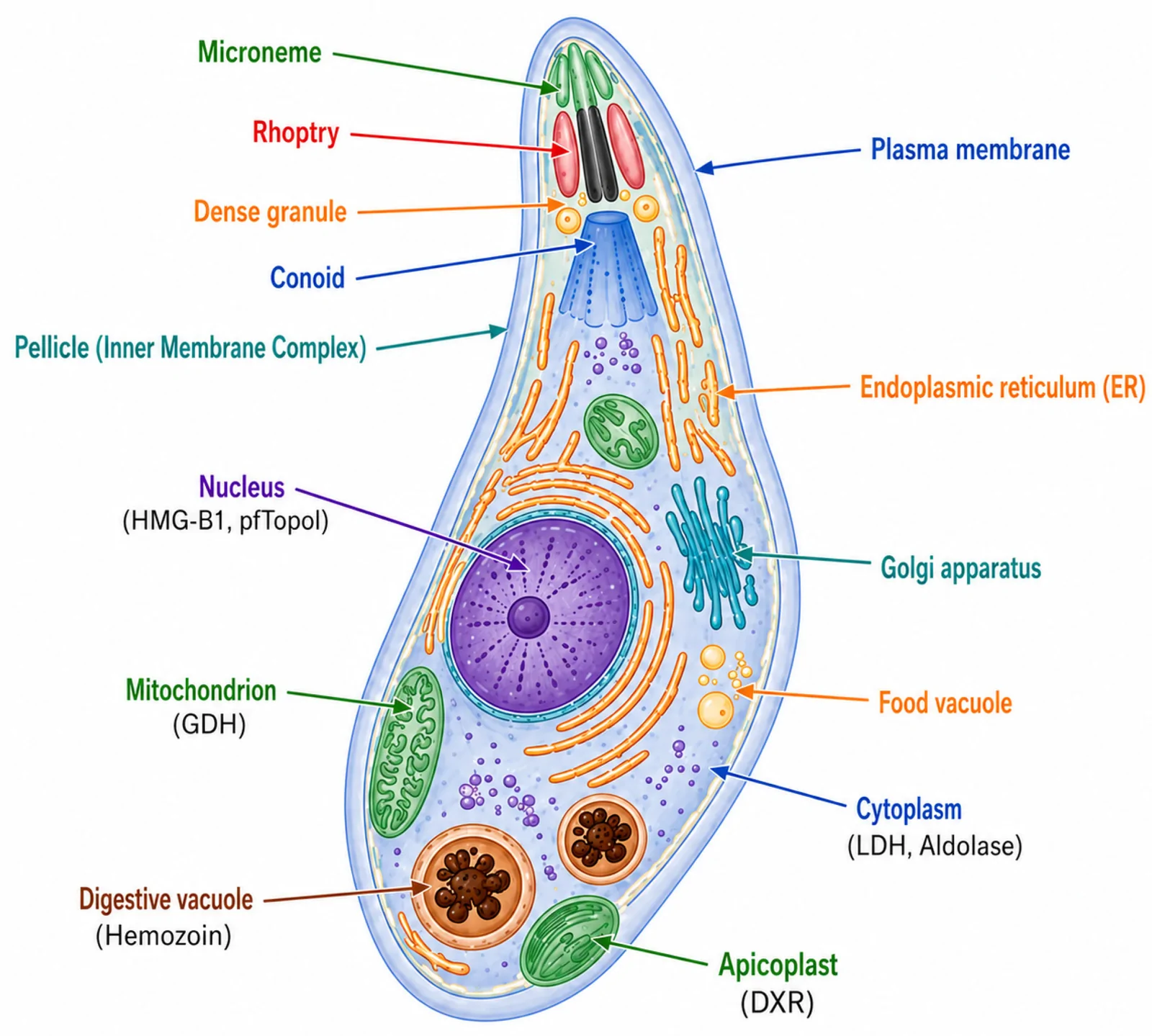
Subcellular localization of malaria biomarkers in *Plasmodium* spp. Illustration of the principal organelles and structural compartments of the malaria parasite, including the apical complex, nucleus, mitochondrion, endoplasmic reticulum, Golgi apparatus, digestive vacuole, food vacuole, apicoplast, cytoplasm, pellicle, and plasma membrane. The apicoplast is an essential plastid organelle involved in metabolic pathways such as isoprenoid precursor biosynthesis and represents an important target for antimalarial research. Main target proteins are shown in parentheses within their subcellular locations: DXR, 1-deoxy-D-xylulose-5-phosphate reductoisomerase; GDH, glutamate dehydrogenase; HMG-B1, high mobility group box 1 protein; LDH, lactate dehydrogenase. The illustration was created using generative AI tools.

Malaria diagnostic biosensors typically target parasite-derived proteins that appear in blood during infection. Among them, the main *Plasmodium* proteins in the serum of an infected patient are lactate dehydrogenase (pLDH; PfLDH/PvLDH), histidine-rich protein II (PfHRP2), glutamate dehydrogenase (PfGDH) and other parasite proteins (e.g., high mobility group box 1 protein, HMGB1). Aptamers have been selected against many of these malaria biomarkers and incorporated into diverse sensor formats (optical, electrochemical, colorimetric, microfluidic), demonstrating the feasibility of aptamer recognition across the principal malaria antigens used for diagnosis [24] (see Section 5 and Section 6). Figure 3 shows a summary of the main biomarkers’ locations in *Plasmodium*.

The main characteristics of the biomarker proteins used for aptamers are described in Table 1.

### 4.1. Lactate Dehydrogenase

Lactate dehydrogenase (E.C. 1.1.1.27.) catalyzes the reversible oxidation of lactate to produce pyruvate using the coenzyme pair NAD+/NADH:

L-lactate + NAD^+^ ⇌ pyruvate + NADH + H^+^

The primary metabolic function of *Plasmodium* LDH (pLDH) is its catalytic role in the glycolytic pathway during the intraerythrocytic stages of the parasite’s life cycle [13]. Because *Plasmodium* parasites are highly dependent on glycolysis for energy production, pLDH is essential for the generation of plasmodial ATP while the parasite resides within human red blood cells [54].

While *Plasmodium* LDH shares a catalytic mechanism with its human counterpart, it serves as a superior diagnostic biomarker due to significant structural and biochemical divergence, most notably a five-residue insertion within the substrate-specific loop. These structural variations—including altered cofactor-binding geometry, distinct kinetic parameters (Km values, lower substrate inhibition), and unique loop-closure dynamics—facilitate a functional divergence that provides essential molecular handles for the development of selective aptasensors (Table 2). These features have been well documented in structural (e.g., crystal structures), biochemical (kinetic assays), and evolutionary (sequence conservation/diversity) studies [13,46,55,56,57].

*Plasmodium* lactate dehydrogenase (pLDH) itself represents a highly valuable biomarker for malaria diagnosis because it is a conserved glycolytic enzyme produced exclusively by metabolically active parasites. Unlike other widely used biomarkers such as histidine-rich protein 2 (PfHRP-2), which may persist in circulation for extended periods after parasite clearance [6,58,59], pLDH is rapidly eliminated, typically within 24 h following successful treatment. This rapid clearance profile makes pLDH a more reliable indicator of active infection and treatment efficacy, thereby increasing its utility in real-time malaria diagnostics and disease monitoring.

Aptamers targeting plasmodial lactate dehydrogenase (pLDH) constitute one of the most extensively studied approaches for malaria biosensing [6,11,58]. Early DNA aptamers developed against PfLDH demonstrated strong selectivity toward the parasitic enzyme over human lactate dehydrogenase (hLDH). Frith et al. reported the generation of two classes of DNA aptamers with high affinity and specificity toward recombinant *Plasmodium falciparum* lactate dehydrogenase (rPfLDH) and a *P. falciparum*-specific lactate dehydrogenase epitope peptide (LDHp) [24]. Sequence analysis identified conserved structural motifs, including the consensus regions GGTAG and GGCG, indicating their central role in target recognition and aptamer folding. Additional motifs, such as ATTAT and polyadenine stretches, were also observed, suggesting further contributions to binding stability and specificity [24]. Among the aptamers selected against the recombinant protein, rLDH4 demonstrated the highest affinity for rPfLDH in ELONA assays, while both rLDH4 and rLDH15 effectively discriminated between *P. falciparum* LDH and *P. vivax* LDH (rPvLDH) [24]. Furthermore, rLDH4 and LDHp11 showed successful in situ binding to *P. falciparum* parasites, as confirmed by confocal microscopy [24].

The LDHp11 aptamer, generated against the species-specific peptide epitope LISDAELEAIFDC, displayed enhanced discriminatory capacity between recombinant PfLDH and PvLDH compared with that of aptamers selected against the full-length recombinant enzyme [24]. This finding demonstrates the utility of epitope-directed aptamer selection strategies for overcoming the high sequence conservation shared among *Plasmodium* LDH isoforms. Consequently, LDHp11 represents a promising biorecognition element for biosensor-based malaria diagnostics capable of differentiating *P. falciparum* and *P. vivax* infections [24,60]. More broadly, these results establish a framework for the rational development of species-specific aptamers targeting unique epitopes from other *Plasmodium* species, thereby supporting the design of highly selective malaria diagnostic platforms [60,61].

To further address the challenge posed by the high structural conservation among pLDH enzymes from different *Plasmodium* species, chemically modified aptamers known as cubamers have also been investigated [47]. These nucleic acids incorporate abiotic cubane moieties, which act as benzene bioisosteres and enhance hydrophobic molecular interactions [47]. Notably, the cubamer 1501s demonstrated the ability to selectively recognize PvLDH over PfLDH despite the strong sequence homology between the two proteins [46]. Binding specificity was attributed to hydrophobic interactions involving the cubane modifications and specific PvLDH residues, particularly Leu232 and Ala233. By integrating directed evolution with synthetic chemical modifications, this strategy achieved excellent analytical sensitivity and low limits of detection within Aptamer-Tethered Enzyme Capture (APTEC)-based diagnostic assays.

Structural studies revealed that this specificity derives from the formation of a distorted hairpin conformation capable of interacting with the extended substrate-specificity loop (residues 102–108) unique to PfLDH [62]. This selective recognition validated the effectiveness of the counter-selection strategy employed during aptamer evolution and enabled the development of sensitive diagnostic platforms, including gold nanoparticle (AuNP)-based colorimetric assays and APTEC systems [61,62,63].

**Table 2:** Comparative features of Plasmodium vs. human lactate dehydrogenases (LDH).

Feature	*Plasmodium* LDH (pLDH)	Human LDH (hLDH)	Functional/Diagnostic Implications	Source Citations
Substrate-specificity loop	A five-amino-acid insertion (e.g., DKEWN) enlarges the loop and changes its flexibility	Lacks the insertion; shorter and more rigid loop.	Enables parasite-specific substrate binding and aptamer/antibody recognition.	[64,65]
Active site geometry	Cofactor (NADH/NAD^+^) binds in a shifted orientation; loop closure is rate-limiting.	Canonical cofactor binding; faster loop closure.	Structural divergence allows for selective inhibitors or aptamers.	[66,67]
Cofactor affinity (Km, NADH)	Km ≈ 7 μM (PfLDH).	Km ≈ 8.3 μM (LDH-H4); 1.3 μM (LDH-M4).	Reflects similar but not identical NADH interactions; exploitable in inhibitor design.	[65,66]
Substrate affinity (Km, pyruvate)	Km ≈ 30 μM (PfLDH).	Km ≈ 60 μM (H4); 180 μM (M4).	Parasite enzyme operates efficiently at lower pyruvate levels.	[65]
Substrate inhibition	Absent or minimal.	Pronounced at high pyruvate concentrations.	Broader linear response range in assays using pLDH.	[64]
Catalytic loop dynamics	Loop closure slower and rate-limiting, influenced by insertion sequence.	Loop closure faster and energetically less demanding.	Distinct kinetics aid species discrimination in enzyme-based biosensors.	[65]
Thermal stability	Exhibits reversible transitions near febrile temperatures (38–42 °C).	More thermally stable; less conformational flexibility.	Adaptation to parasite’s intraerythrocytic conditions; potential biosensor parameter.	[68]
Epitope/antigenicity	Contains unique structural epitopes absent in hLDH.	Highly conserved among mammalian LDHs.	Enables selective detection by aptamers/antibodies.	[69,70]
Genetic conservation	*ldh* gene conserved among *Plasmodium* spp.; loop insertion universally retained.	Multiple human isoforms (LDH-A, -B, -C) encoded by distinct genes.	Facilitates genus-specific diagnostics and cross-species comparisons.	[71]

### 4.2. Glutamate Dehydrogenase

Glutamate dehydrogenase (GDH) catalyses the reversible oxidative deamination of L-glutamate to α-ketoglutarate and ammonia, reducing NAD(P)^+^ to NAD(P)H:

L-Glutamate + NAD(P)^+^ + H_2_O ⇌ α-ketoglutarate + NH_4_^+^ + NAD(P)H + H^+^

*Plasmodium falciparum* glutamate dehydrogenase (PfGDH) has emerged as a promising biomarker for malaria diagnostics due to its essential role in parasite nitrogen metabolism and its differential structural features compared with the human homolog. PfGDH is expressed during the erythrocytic stages of infection and is associated with metabolically active parasites, making it a relevant target for the detection of active malaria infections.

In *Plasmodium falciparum*, there are multiple GDH isoforms (e.g., PfGDH1, PfGDH2, PfGDH3) that are NADP-dependent and play roles in redox metabolism and possibly in organellar differential localization [72]. Human GDH (e.g., hGDH1, hGDH2) is also hexameric but has distinct regulatory features, allosteric control, dual cofactor specificity, and is embedded in different tissue and subcellular contexts [73].

Key differences between PfGDH and human GDH are shown in Table 3. Whereas human GDH1 and GDH2 often have dual specificity for NAD^+^ and NADP^+^ (or at least flexibility depending on physiological conditions) [73], PfGDH is predominantly NADP(H)-dependent. *P. falciparum* GDHs (at least PfGDH1 and PfGDH2) require NADP (i.e., NADP^+^/NADPH) as a coenzyme in their oxidative deamination/reductive amination reactions [72].

The PfGDH enzymes are hexameric. Between PfGDH1 and PfGDH2, there are differences in the inter-subunit interfaces: PfGDH1 shows stronger networks of salt bridges mediating subunit contacts, whereas PfGDH2 (and mammalian GDHs) have more hydrophobic interactions. PfGDH also has a unique N-terminal extension that is absent in mammalian GDH sequences [74]. Human GDH is also homohexameric, but the subunit interfaces are different: They are more hydrophobic (fewer salt bridges) and lack the extra parasite-specific N-terminal extension. Moreover, human GDH has regulatory “antenna” or “pivot helix” regions associated with allosteric regulation (by ADP or GTP) [75].

Regarding the kinetic parameters, PfGDH1 and PfGDH2 have similar specific activities, but PfGDH2 shows slightly higher $K_{m}$ values for substrates. This suggests that PfGDH2 binds substrate less tightly than PfGDH1 [72]. Human GDHs differ among themselves (GDH1 vs. GDH2) in kinetics: They have different $K_{m}$ values for glutamate, for 2-oxoglutarate, and for ammonium; they also have different optimum pH values and regulation by nucleotides (ADP, GTP) [76].

There is less known regulatory complexity in PfGDH. Structural studies suggest that PfGDH may lack some of the classic allosteric regulatory sites of mammalian GDH or differ in how those sites are configured. The parasite enzyme may be more constitutively active, especially for maintaining NADPH pools and responding to oxidative stress [74]. Human GDH is strongly allosterically regulated. It is sensitive to ADP (activator), GTP (inhibitor), and other metabolites; conformational changes are induced by these effectors; regulation is fine-tuned for metabolic demands, tissue specificity, and energy status [75].

The different PfGDH isoforms have different subcellular localizations, e.g., PfGDH2 localizes to the apicoplast, whereas PfGDH1 and PfGDH3 are cytosolic. Their roles include contributions to redox balance (via NADPH production) and amino acid metabolism, possibly including feedback under stress [72]. In human cells, GDH (hGDH) is mitochondrial, is largely involved in amino acid catabolism, links to the Krebs cycle, and in tissues such as the liver, brain, and kidney.it also plays roles in the regulation of ammonia levels, neurotransmitter metabolism (in the brain), etc. [73].

In recent years, nucleic acid aptamers directed against PfGDH have been investigated as alternative biorecognition elements to antibodies because of their high specificity, thermal stability, low production cost, and ease of chemical modification.

SELEX-based selection strategies have successfully generated DNA aptamers capable of binding recombinant PfGDH with high affinity and selectivity. Thus, NG3, a thiolated ssDNA aptamer specific for *P. falciparum* GDH, was selected via 17 rounds of SELEX [52] and exhibited a dissociation constant of 79.16 ± 1.58 nM. This aptamer demonstrated limited cross-reactivity with human glutamate dehydrogenase, indicating its potential suitability for species-specific malaria diagnostics. Structural analyses suggested that aptamer recognition is mediated through the formation of stable secondary structures, including stem-loop and hairpin motifs that interact with exposed parasite-specific regions of the enzyme [13,52]. Due to their robust binding characteristics, PfGDH-targeting aptamers have been proposed for integration into electrochemical and optical biosensing platforms aimed at rapid malaria diagnosis in point-of-care settings [77].

The diagnostic relevance of PfGDH is further supported by its intracellular abundance and its persistence during active parasite metabolism. Compared with conventional antibody-based assays, aptamer-based systems targeting PfGDH offer advantages in assay reproducibility, storage stability, and adaptability to miniaturized biosensor technologies. Consequently, PfGDH aptamers represent promising candidates for the development of next-generation malaria diagnostic devices with improved sensitivity and operational stability under field conditions [13,52].

### 4.3. Aldolase

Aldolase participates in the glycolytic pathway of *Plasmodium* parasites and is expressed across multiple species, including *P. falciparum* and *P. vivax*. Because of its conservation among human-infective *Plasmodium* species, aldolase has traditionally been used as a pan-malarial diagnostic marker in RDTs. However, limitations associated with antibody-based detection, including antigen instability and variable assay sensitivity, have motivated the exploration of aptamer-based recognition systems.

To the best of our knowledge, no aptamer specific for aldolase has been obtained. Aldolase-targeting aptamers could have considerable potential for incorporation into biosensor platforms, including fluorescence-based, electrochemical, and nanoparticle-assisted detection systems. Their ability to recognize conserved aldolase epitopes could enable broad-spectrum malaria detection, making them particularly suitable for pan-species diagnostic applications. Furthermore, the chemical stability and synthetic accessibility of aptamers could provide advantages over monoclonal antibodies in terms of manufacturing scalability, storage conditions, and compatibility with portable diagnostic technologies [78].

Overall, aptamers directed against plasmodial aldolase would represent useful molecular tools for malaria diagnostics. Their integration into biosensor platforms, similar to the ones using antibodies [78], would contribute to the development of highly sensitive, stable, and field-deployable diagnostic systems capable of improving malaria surveillance, early detection, and therapeutic monitoring.

### 4.4. Plasmodium Falciparum Histidine-Rich Protein 2 (PfHRP-2)

PfHRP-2 is one of the most extensively used biomarkers for the diagnosis of *Plasmodium falciparum* malaria. This water-soluble protein is secreted by asexual blood-stage parasites and released into the bloodstream during the rupture of infected erythrocytes. Owing to its high abundance and relative stability in circulation, PfHRP-2 has become a primary target in RDTs. However, several limitations associated with antibody-based detection systems, including thermal instability, batch-to-batch variability, and high production costs, have driven the search for alternative molecular recognition elements. Among these, aptamers have emerged as promising candidates.

Unlike other metabolic biomarkers, PfHRP2 is a non-enzymatic protein whose unique antigenicity stems from a lack of any structural or sequence-based human equivalent [79]. While its physiological roles in heme detoxification and immune modulation remain subjects of ongoing research, its abundance and specific secretion by the trophozoite stage into the bloodstream make it a cornerstone of current *P. falciparum* diagnostics.

Due to its differences from human histidine-rich proteins, PfHRP2 plays a diagnostic role in malaria detection. It is synthesized by the trophozoite stage of *P. falciparum* and is released into the host’s bloodstream as the parasite develops and ruptures infected red blood cells. Its diagnostic importance lies in its abundance, species specificity, and detectability in blood, though challenges with gene deletions and antigen persistence continue to affect test reliability.

PfHRP2 is characterized by a high proportion of histidine and alanine residues, frequently arranged in repetitive motifs. In many isolates, the protein contains many repeat units (such as “AHH” or “AHHAAD”) [80]. These repetitive histidine-rich regions are rare in human proteins; there is no human homolog of HRP2 with the same kind of histidine-alanine repeat architecture. The protein is expressed by *P. falciparum* during the asexual blood stages, released into the infected erythrocyte cytoplasm, and can be found circulating in the host’s peripheral blood. It is secreted (or released) upon schizont rupture, contributing to the antigen pool in plasma. The human bloodstream has many proteins but none with similar features to those of HRP2 (see Table 4).

There is extensive sequence variation in the *pfhrp2* gene among *P. falciparum* isolates globally. The variation arises from differences in the number, order, and type of repeat units. These polymorphisms can affect the antigenic epitopes recognized by antibodies in RDTs [81]. Some *P. falciparum* strains completely lack the *pfhrp2* gene (and in some cases *pfhrp3*, a paralog) due to gene deletion, especially in certain geographic regions. Deletions of *pfhrp2* cause false negatives in HRP2-based RDTs [82].

**Table 4:** Comparative points between PfHRP2 and human proteins.

Feature	PfHRP2 (*Plasmodium falciparum* Histidine-Rich Protein II)	Human Counterpart/ Human Proteins	Implications for Diagnostics & Biosensor Design
Amino acid composition	Very high %: His (~34%), Ala (~37%), Asp (~10%); many repetitive motifs (e.g., “AHH”, “AHHAAD”) in exon 2; repeat types vary in number & order [80].	Human proteins typically have more varied, non-repetitive composition; some histidine residues in many proteins but not in large repetitive histidine-alanine repeat units like HRP2.	The unusual repetitive motifs create strong antigenic surfaces that can be targeted; variation in repeats affects binding/epitope availability.
Genetic variation/polymorphism	High variability in repeat number and arrangement; exon 2 size varies widely; gene deletions occur in some parasite populations [80].	Human genome is relatively stable; no equivalent gene with similar variability in healthy populations for such a repetitive antigen.	Diagnostic tests using HRP2 must account for variation; engineered aptamers or antibodies should target conserved repeat units; gene deletion surveillance is vital.
Specificity to the parasite	HRP2 is specific to *P. falciparum*. Expression consistent across blood stages; released into the bloodstream [80].	Humans lack HRP2, and similar histidine-rich proteins are rare, resulting in high target specificity.	Provides high assay specificity; enables low cross-reactivity; ideal for diagnostics if antigen is present.
Diagnostic challenges	*pfhrp2/pfhrp3* deletions cause false-negative HRP2-RDTs; repeat and geographic variation reduce detection sensitivity [82].	Low interference risk due to stable human proteins; however, non-specific binding, anti-histidine antibodies, and background binding must be controlled.	Biosensor or immunoassay must include appropriate controls, highly specific antibodies/aptamers, and multiplexing (e.g., with pLDH) to overcome HRP2 deletion and variability.
Expression/ abundance	Abundant in infected erythrocytes and circulation; accumulation makes HRP2 a highly sensitive biomarker [79].	Equivalent human proteins are present but with very different contexts; no human antigen that accumulates similarly in malaria infection.	High abundance enhances sensitivity of detection (lower LODs possible); useful for RDTs and potentially aptamer sensors.

No known human protein has both the repetitive histidine-rich motif structure and the same antigenic features as PfHRP2. Thus, antibodies or aptamers against PfHRP2 have high specificity (provided the epitope is conserved) and low cross-reactivity with human antigens. While human blood contains many proteins, the histidine-rich repeats are unusual. However, human serum metal-binding proteins, histidine-rich peptides, or histidine residues in human proteins could theoretically cause non-specific binding in poorly designed assays. Good assay design and counter-selection are necessary.

Because PfHRP2 is abundant, released into blood, and specific to *P. falciparum*, it has been widely used as an antigen target in RDTs for *P. falciparum* malaria. Its repetitive histidine-rich segments allow strong binding by anti-HRP2 antibodies [80]. The variability in repeat types affects detection sensitivity at low parasite densities. Isolates with fewer repeats or certain patterns may be less well detected. In addition, strains lacking *pfhrp2* are not detected by HRP2-based RDTs [81]. HRP3 is a paralogous protein encoded by *pfhrp3* that shares many repeat motifs with HRP2 and can cross-react with anti-HRP2 antibodies. This can sometimes compensate (to a degree) for pfhrp2 deletion in detection, depending on RDT design [80].

Several DNA aptamers targeting PfHRP-2 have been developed for malaria biosensing applications (B4, 2106s) [83] and typically exhibit dissociation constants in the nanomolar range, indicative of strong binding affinity. The biochemical characteristics of PfHRP-2, including its high histidine and alanine content, repetitive amino acid motifs, intrinsically disordered structure, and overall positive charge under physiological conditions, facilitate electrostatic and π-interactions with guanine-rich aptamer sequences, particularly those capable of forming G-quadruplex structures. Compared with monoclonal antibodies, aptamers offer several advantages, such as superior thermal stability, high reproducibility through chemical synthesis, extended shelf life, lower production costs, and straightforward chemical modification. These properties make aptamers particularly attractive recognition elements for the development of robust point-of-care diagnostic devices suitable for resource-limited malaria-endemic regions [25,26,27,28].

Anti-PfHRP-2 aptamers have been incorporated into a variety of biosensing platforms, including electrochemical aptasensors, surface plasmon resonance (SPR) systems, fluorescence-based sensors, lateral flow assays, and nanoparticle-assisted detection strategies. Among these approaches, electrochemical aptasensors have demonstrated particularly high analytical sensitivity, with some platforms achieving picomolar limits of detection for recombinant PfHRP-2. Despite these promising results, several challenges remain before widespread clinical implementation can be achieved. These include the occurrence of *pfhrp2* gene deletions in certain *P. falciparum* strains, which may lead to false-negative diagnostic results, potential cross-reactivity with the structurally related PfHRP-3 protein, matrix effects caused by complex blood components, and the limited number of large-scale clinical validation studies. Current research efforts are focused on the development of next-generation high-affinity aptamers, multiplexed malaria biosensors, microfluidic-integrated platforms, smartphone-assisted detection technologies, and hybrid aptamer–nanomaterial architectures [84,85]. In addition, machine learning-assisted SELEX strategies and advanced structural modeling are emerging as powerful tools for optimizing aptamer selection and enhancing diagnostic performance.

### 4.5. DNA-Binding Proteins: HMGB1

HMGB1 is a non-histone chromatin-binding protein. There are also some differences between HMGB1 in *Plasmodium* and in humans (see Table 5). In humans (and other mammals), HMGB1 has multiple domains: two DNA-binding domains (“A-box” and “B-box”), a C-terminal acidic tail, and various regulatory cysteines; it functions both in the nucleus (DNA repair, transcriptional regulation, chromatin remodelling) and extracellularly as a damage-associated molecular pattern (DAMP), participating in inflammation through receptors like TLR4 and RAGE [86,87]. *Plasmodium falciparum* also encodes HMGB1 (PfHMGB1), though its structure is more minimal/divergent. Computational and experimental studies show that PfHMGB1 is shorter (≈ 71–91 amino acids in many analyses), contains a single HMG box (more similar to the B-box of human HMGB1), lacks some domains found in human HMGB1, and functions largely in the nucleus, particularly in asexual erythrocytic stages of the parasite. PfHMGB1 is expressed through many parasite stages and is capable of bending DNA and binding distorted DNA structures [88].

PfHMGB1 is proposed as a robust biomarker due to its high expression across all *P. falciparum* blood stages, high stability, and low identity (39.4% identity) to its human homolog (HMG-box). Aptamers like PfR6 developed against the HMG-box domain achieved high binding affinity and were shown to bind rapidly in stopped-flow assays (less than one minute), making them suitable candidates for RDT development. These characteristics have motivated the development of nucleic acid aptamers specifically targeting PfHMGB1 for diagnostic applications. Another aptamer, PfE3, shows a lower affinity (nM) but demonstrates the greatest thermophoretic amplitude in binding assays [52].

Recent studies employing SELEX successfully generated DNA aptamers with high affinity and specificity toward recombinant PfHMGB1. Counter-selection procedures against human HMGB1 were incorporated during the selection process to minimize cross-reactivity and improve parasite specificity. The resulting aptamers demonstrated nanomolar-range binding affinities and selective recognition of PfHMGB1 over the human protein, highlighting their potential as highly discriminative biorecognition elements [52,88].

Structural and biophysical analyses revealed that PfHMGB1-binding aptamers adopt stable secondary conformations, including stem-loop and hairpin architectures, that facilitate target recognition through complementary electrostatic and hydrophobic interactions. Several aptamers also exhibited conformational rearrangements upon target binding, suggesting induced-fit recognition mechanisms that contribute to high-affinity complex formation. Importantly, these aptamers retained functionality under physiological conditions, supporting their suitability for integration into diagnostic biosensors [89].

PfHMGB1-targeting aptamers have been evaluated in multiple biosensing configurations, including electrochemical, fluorescence-based, and surface plasmon resonance (SPR) platforms. Their high sensitivity enabled the detection of low concentrations of recombinant PfHMGB1 and parasite-derived antigens, indicating potential utility for early-stage malaria diagnosis. Compared with conventional antibody-based assays, aptamer-based PfHMGB1 detection systems offer several advantages, including improved thermal stability, reduced production costs, ease of chemical synthesis, and compatibility with portable point-of-care devices [52,90].

The diagnostic relevance of PfHMGB1 is particularly significant because the protein is associated with active parasite metabolism and may be released during parasite replication and host–cell rupture. Unlike antigens such as PfHRP-2, whose persistence in circulation can lead to false-positive results after parasite clearance, PfHMGB1 has been proposed as a more dynamic biomarker reflecting ongoing infection. Consequently, aptamers targeting PfHMGB1 may contribute to the development of highly sensitive and species-specific malaria diagnostic technologies capable of improving infection monitoring and treatment assessment [52,91].

Overall, PfHMGB1-directed aptamers represent an emerging class of molecular recognition elements with considerable potential for next-generation malaria biosensors. Their combination of specificity, stability, and adaptability positions them as attractive alternatives to antibodies for the development of rapid and accurate detection systems.

**Table 5:** Comparative features of Plasmodium and human non-histone chromatin-binding proteins (HMGB).

Feature	PfHMGB1 (*Plasmodium*)	Human HMGB1
Length & domain composition	Single HMG box domain (~71–91 aa), lacks A-box and C-terminal acidic tail present in humans; no tandem boxes [92].	Two HMG boxes (A-box and B-box), plus acidic C-terminal tail (~30 residues), with nuclear localization signals [86].
Key cysteine residues (redox- sensitive)	Lacks key cysteines found in human HMGB1 (Cys23, Cys45, and Cys106); Cys106 is replaced by Ala [93].	Contains three conserved cysteines (Cys23, Cys45 from A-Box, Cys106 from B-Box) whose redox states regulate cytokine activity and nuclear localization [86].
Sequence identity/similarity	Highly conserved among human-infecting *Plasmodium* species (≈80–90% identity), but only ~39% identical to the human HMG-box [52].	High conservation among mammals (≈98.5–99%) in HMGB1; sequence nearly identical across vertebrates [94].
Functional domain absence	Lacks the anti-inflammatory A-box and acidic tail; does not induce TNF-α or other pro-inflammatory responses like human HMGB1 [93].	Has both pro- and anti-inflammatory roles (B-box, A-box), extracellular activities, DAMP roles, mediated via receptors like TLR4/MD-2, RAGE; redox regulation via cysteines [95].

### 4.6. Apicoplast Enzymes and Isoprenoid Pathway

The methylerythritol phosphate (MEP) pathway, also known as the non-mevalonate pathway, is an essential metabolic route responsible for the biosynthesis of isoprenoids in many bacteria, plant plastids, and apicomplexan parasites such as *Plasmodium falciparum*. In *Plasmodium* species, the pathway is localized within the apicoplast, a relict plastid organelle derived from secondary endosymbiosis. Because humans lack the MEP pathway and instead synthesize isoprenoids through the mevalonate pathway, the *Plasmodium* MEP pathway has emerged as a highly attractive target for antimalarial drug development. Its biological importance, unique evolutionary origin, and absence in mammalian hosts make it central both to parasite survival and to therapeutic research [96,97,98,99].

Isoprenoids constitute one of the largest and most diverse classes of biomolecules in nature. They include compounds involved in electron transport, membrane integrity, protein prenylation, antioxidant defense, and hormone synthesis. In *Plasmodium*, isoprenoid products are required for multiple vital cellular processes, including ubiquinone biosynthesis, dolichol production, tRNA modification, and prenylation of proteins involved in intracellular trafficking and development. The importance of the pathway is demonstrated by the fact that inhibition of MEP pathway enzymes arrests parasite growth and leads to parasite death [96,100].

The pathway takes place in the apicoplast, a non-photosynthetic plastid surrounded by four membranes. Although the apicoplast no longer performs photosynthesis, it retains several anabolic functions essential for parasite viability, including fatty acid synthesis, iron-sulfur cluster assembly, and isoprenoid precursor biosynthesis. Among these functions, isoprenoid biosynthesis appears to be the only universally essential role during the blood stages of *Plasmodium* infection. Supplementation with isopentenyl pyrophosphate (IPP), the end product of the pathway, can rescue parasites from apicoplast loss, demonstrating that IPP synthesis is the critical indispensable function of the organelle during erythrocytic growth [101].

The pathway begins with the condensation of pyruvate and glyceraldehyde-3-phosphate (G3P). This first committed step is catalyzed by 1-deoxy-D-xylulose-5-phosphate synthase (DXS), producing 1-deoxy-D-xylulose-5-phosphate (DXP). DXS is considered a regulatory enzyme because it controls carbon flux into the pathway. DXP serves not only as a precursor for isoprenoids but also participates in the biosynthesis of vitamins such as thiamine and pyridoxal in other organisms.

The second step is catalyzed by DXP reductoisomerase (DXR), also known as IspC, which converts DXP into 2-C-methyl-D-erythritol-4-phosphate (MEP). This reaction requires NADPH and divalent metal ions. DXR is one of the most extensively studied enzymes of the pathway because it is inhibited by fosmidomycin, a phosphonic acid antibiotic with potent antimalarial activity [96].

Roca et al. [51] developed a DNA aptamer (D10) that binds in vitro to recombinant DXR from *P. falciparum* and *Escherichia coli*. According to fluorescence confocal microscopy data, this aptamer specifically targets the apicoplast organelle in *P. falciparum* in vitro cultures where the MEP pathway is localized and is, therefore, a highly specific marker of red blood cells parasitized by Plasmodium vs. naïve erythrocytes.

6FAM-labeled D10 specifically discriminated non-parasitized red blood cells from *Plasmodium*-infected RBCs, according to flow cytometry. The confocal fluorescence microscopy data on the subcellular targeting of 6FAM-labeled D10 in pRBCs showed a punctate pattern consistent with apicoplast localization. D10 was observed to bind all the blood stages of the parasite, including ring forms, in agreement with targeting the apicoplast, as this organelle is present in all the intraerythrocytic forms of the pathogen. Targeting the apicoplast was further confirmed by fluorescence confocal microscopy through colocalization analysis with an antibody against the apicoplast enzyme ferredoxin-NADP reductase (FNR) [51].

For malaria research, the study of *Plasmodium* organelles, dynamic processes and changes in the metabolism of the parasite is of great importance. Visualization of the pathogen and its subcellular compartments greatly contributes to understanding its biology [102]. Few apicoplast markers are currently available, and to the best of our knowledge, all of them rely on the use of antibodies, e.g., against FNR [103,104] and the acyl carrier protein [105,106], or of apicoplast-targeted green fluorescent protein [107]. In this regard, the apicoplast-specific D10 aptamer tagged with fluorescent molecules or biotin represents an important addition to the small toolkit for the investigation of the cellular biology of malaria parasites.

In summary, aptamer D10, targeting 1-deoxy-D-xylulose-5-phosphate reductoisomerase (DXR), an essential enzyme in the isoprenoid biosynthesis pathway, is a specific marker of *Plasmodium*-infected erythrocytes and of the apicoplast organelle, presenting this aptamer as a potential element of future malaria diagnostic strategies and as a valuable tool for cellular biology studies in *Plasmodium* and other Apicomplexa DNA [51].

Aptamers specific for end products of the isoprenoid pathway in malaria parasites have also been prepared. For example, AptPP is a DNA aptamer developed through SELEX that specifically binds linear cis- and trans-polyisoprenoids containing oxygenated α-isoprene units, such as dolichol and polyprenal, while showing limited affinity for unrelated lipids and metabolites. The aptamer displayed higher affinity for dolichol than for dolichyl phosphate or nor-dolichol, indicating that both the α-isoprene unit and the polyisoprenoid chain contribute to molecular recognition. Sequence analysis identified a conserved motif (ATGTCGACTG) that contributes to the structural stability and binding specificity of the aptamer. Structural predictions using the mFold web server revealed that AptPP adopts a stable secondary structure essential for target recognition [53].

Fluorescently labeled AptPP variants (6-FAM-AptPP and Cy5-AptPP) were successfully applied for in situ imaging of polyisoprenoids throughout the intraerythrocytic developmental stages of *P. falciparum*. Confocal microscopy studies revealed dynamic subcellular localization patterns, including partial colocalization with the endoplasmic reticulum, apicoplast, Golgi, and mitochondrial markers. Furthermore, changes in AptPP localization following genetic knockdown of polyprenol reductase (PfPPRD) and chemical inhibition of isoprenoid biosynthesis confirmed its specificity for polyisoprenoid metabolites. These findings establish AptPP as a promising molecular tool for studying isoprenoid metabolism and intracellular lipid distribution in malaria parasites [53].

### 4.7. Heme Pathways

The first report on *Plasmodium*-specific aptamers dates from 2009, when Barfod et al. successfully isolated a specific RNA aptamer against the DBL1α region of the *P. falciparum* erythrocyte membrane protein 1, which is associated with heme metabolism and parasitized RBC (pRBC) adhesion to blood vessels and erythrocytes [108]. Aptamers binding the DBL-1α domain, b02, d12 and e05, have been described for PfEMP1 [108]. In the same year, Niles et al. isolated a DNA aptamer that specifically bound the heme group and inhibited hemozoin formation in vitro in a similar way to that of the antimalarial compound chloroquine. Consistent loading of this aptamer into RBCs resulted in a reduction in Plasmodium viability due to the accumulation of toxic heme in the pRBCs [109].

Hemozoin is a crystalline by-product formed during the intraerythrocytic stage of *Plasmodium* infection. As the parasite digests host hemoglobin within its digestive vacuole, toxic free heme is released and subsequently detoxified through its conversion into insoluble hemozoin crystals. Because hemozoin is produced exclusively by malaria parasites and accumulates proportionally with parasite development, it represents a highly specific biomarker for malaria diagnosis [13,16,21].

Unlike protein-based biomarkers such as HRP2 or pLDH, hemozoin detection does not rely on antibodies or nucleic acid amplification. Instead, diagnostic approaches exploit its unique optical, magnetic, and spectroscopic properties. Hemozoin crystals exhibit strong Raman signatures that can be amplified using surface-enhanced Raman scattering (SERS), enabling highly sensitive detection at very low parasitemia levels. In addition, the crystals possess paramagnetic properties due to their iron-containing structure, allowing detection through techniques such as magnetic resonance relaxometry (MRR), which measures alterations in proton relaxation times caused by the presence of hemozoin-containing infected erythrocytes [6,16,21].

The amount of hemozoin produced increases throughout parasite maturation, making it a useful indicator of parasite burden and infection progression. Furthermore, because hemozoin remains detectable in infected red blood cells without requiring labeling reagents, it is particularly attractive for the development of rapid, low-cost, and reagent-free diagnostic platforms. However, residual hemozoin may persist after parasite clearance, which can complicate the distinction between active and recently resolved infections in some settings [13,16,21].

There are currently very few true aptasensors specifically designed for hemozoin detection, and most malaria aptasensor research has instead focused on the previously described protein biomarkers such as pLDH, PfHRP-2, and PfGDH. However, aptamers that bind heme or interfere with hemozoin formation have been reported, and these studies provide an important conceptual basis for future hemozoin-directed aptasensors. Early work by Niles et al. [109] demonstrated that DNA aptamers (PS2R, PS2M, PS21, PS26, OKA26-3, OKA-26-5) could specifically bind free heme and inhibit hemozoin crystallization in vitro, producing antimalarial effects similar to those of chloroquine (CQ) through accumulation of toxic heme species inside infected erythrocytes [110]. In contrast, most current hemozoin detection systems are not aptamer-based but instead exploit the unique physicochemical properties of hemozoin crystals, including their magnetic, optical, Raman-scattering, and birefringent characteristics. Techniques such as surface-enhanced Raman scattering (SERS), MRR, polarized light microscopy, and magnetic sensing platforms have demonstrated highly sensitive hemozoin detection without requiring antibodies or aptamers [111].

### 4.8. Plasmodium falciparum DNA Topoisomerase I (pfTopoI)

*Plasmodium falciparum* DNA topoisomerase I (pfTopoI, also referred to as pTOP1) is an essential parasite enzyme involved in DNA replication, transcription, and chromosome maintenance. Unlike conventional malaria biomarkers that detect parasite proteins or nucleic acids, pTOP1-based assays measure the enzymatic activity of a parasite-specific target, providing direct evidence of the presence of viable parasites [5,112]. Because the assay relies on catalytic activity rather than simple biomarker abundance, signal amplification is achieved through enzyme turnover, contributing to high analytical sensitivity.

The Rolling Circle-Enhanced Enzyme Activity Detection (REEAD) platform exploits the ability of pfTopoI to specifically cleave and religate a synthetic DNA substrate engineered into a dumbbell-shaped structure. Upon enzymatic processing, the substrate is converted into a closed circular DNA molecule that serves as a template for rolling circle amplification (RCA). The resulting long DNA products are subsequently detected through fluorescent, colorimetric, or other readout methods, enabling highly sensitive quantification of pfTopoI activity [112].

A major advantage of the REEAD system is its high specificity for malaria parasites, as the DNA substrate can be designed to preferentially interact with the parasite enzyme while minimizing interference from human topoisomerases. Furthermore, because enzyme activity rapidly decreases after parasite death, pTOP1-based detection may provide information on parasite viability and treatment response, potentially distinguishing active infections from residual parasite material that can remain detectable by nucleic acid-based methods [5].

Recent developments have focused on simplifying the assay for point-of-care use through microfluidic integration, portable detection formats, and reduced processing requirements. These improvements highlight the potential of pTOP1 activity assays as highly sensitive tools for malaria diagnosis, treatment monitoring, and surveillance in low-transmission settings where conventional diagnostic methods may lack sufficient sensitivity [5,112].

### 4.9. Other Biomarkers

Apart from using well-characterized malaria biomarkers such as PfHRP2, pLDH, or PfGDH as molecular targets for aptamer development, it is also possible to generate highly specific aptamers through Cell-SELEX using infected and non-infected human red blood cells as selection platforms. In this context, Lantero et al. [50], using a synthetic ssDNA library containing approximately 10^14^ unique sequences with randomized 40-nucleotide regions, performed ten iterative enrichment cycles involving negative selection against non-infected erythrocytes and positive selection against fixed *Plasmodium falciparum*-infected red blood cells. This strategy enabled the isolation of five main aptamer candidates (19, 24, 30, 77, and 78) with remarkable specificity, exhibiting binding rates above 90% for infected erythrocytes and less than 0.1% for healthy cells. Importantly, these aptamers target stable intracellular parasite epitopes rather than highly variable surface antigens, thereby reducing the risk of diagnostic failure caused by antigenic variation or gene deletions. Their G-rich sequences likely promote G-quadruplex formation, thereby enhancing structural stability and affinity for parasite proteins. Clinically, these aptamers demonstrated pan-malaria detection capacity, recognizing not only *P. falciparum* but also *P. vivax*, *P. ovale*, and *P. malariae*, including low-density and asymptomatic infections containing transmissible gametocytes [50]. Consequently, Cell-SELEX-derived aptamers constitute promising, robust, and cost-effective components for next-generation malaria diagnostic platforms aimed at supporting global malaria elimination strategies.

Although most aptamer-based malaria biosensors have focused on biomarkers associated with asexual blood stages, such as PfHRP2, PfLDH, PfGDH, or parasite nucleic acids, the detection of transmission stages remains an important unmet need. Malaria elimination strategies require not only the diagnosis of symptomatic infections but also the identification of individuals carrying mature gametocytes, which are responsible for parasite transmission to mosquitoes and often persist at low densities in asymptomatic carriers. At present, no aptamers have been reported against well-established gametocyte-specific biomarkers, such as Pfs25, Pfs230, or other stage-specific antigens, representing a significant gap in the field. The development of high-affinity aptamers targeting gametocyte-specific molecules would enable the design of multiplexed biosensors capable of simultaneously detecting asexual-stage biomarkers together with transmission-stage markers. Such platforms could distinguish active clinical infections from transmission reservoirs, providing valuable information for surveillance, targeted interventions, and malaria elimination programs. Therefore, the identification of aptamers against gametocyte-specific targets should be considered a priority for the next generation of malaria biosensors [113,114,115].

## 5. Types of Biosensors Employing Aptamers (Aptasensors)

**Figure 4:**
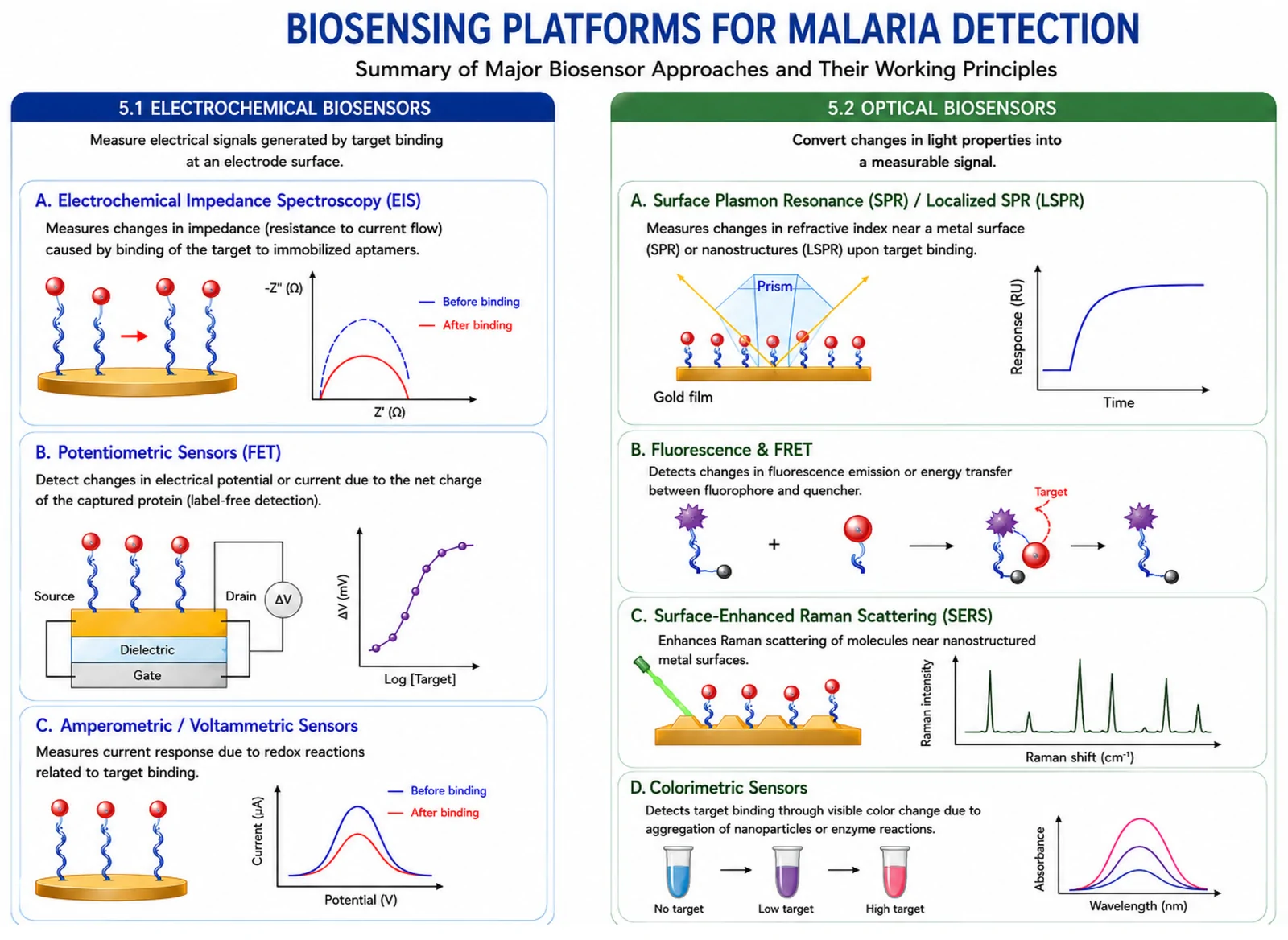
Schematic overview of biosensor transduction strategies applied to malaria diagnostics. Electrochemical biosensors detect analyte binding through changes in electrical properties, including impedance (EIS) and potential/current variations in field-effect transistor (FET)- and amperometric/voltametric-based sensors. Optical biosensors convert molecular interactions into measurable changes in refractive index (SPR/LSPR), fluorescence or Förster resonance energy transfer (FRET), Raman scattering signals (SERS) and colorimetric sensors. Created by the author based on [6,13,16,36,37,38,42,48,117,118]. The illustration was created using generative AI tools.

Aptamers are highly adaptable to various transducer technologies, providing high performance in miniaturized platforms suitable for POC applications [36,38]. Biosensors use several methods to detect aptamers, such as electrochemical, optical, and magnetic methods, which are the most commonly used. A summary of the most commonly used biosensors is shown in Figure 4.

### 5.1. Electrochemical Biosensors

Electrochemical biosensors measure electrical signals—such as current, potential, or impedance—generated upon the interaction between the analyte and the bioreceptor immobilized on an electrode surface [6,38,42,48]. They offer high sensitivity, low cost, and fast response times [6,38,116].

Electrochemical Impedance Spectroscopy (EIS) measures changes in electrical impedance (resistance to current flow) resulting from molecular binding events on the electrode surface [6,38,42,48,116]. Impedance changes correlate with alterations in surface charge distribution or electron transfer kinetics when targets bind to immobilized aptamers [42,116]. EIS aptasensors often achieve low detection limits, sometimes in the femtomolar (fM) range [13]. The aptamer NG3 targeting PfGDH utilized an EIS measurement with a dynamic range of 100 fM to 100 nM [13].

Potentiometric Sensors (Field-Effect Transistors (FETs)) detect changes in electrical potential or current resulting from the intrinsic net charge of the captured protein (label-free detection) [13,117]. The aptamer NG3, targeting PfGDH, was successfully integrated into an Extended Gate FET (EgFET) device immobilized on gold microelectrodes, achieving a linear detection range of 100 fM–10 nM in serum samples [13,117]. The small size of folded aptamers helps overcome the Debye length limitation, thereby maximizing charge-sensing potential in FET measurements [117].

### 5.2. Optical Biosensors

Optical biosensors convert changes in light properties (e.g., absorption, reflection, fluorescence) resulting from the analyte-bioreceptor interaction into a measurable signal [6,16,42,118].

Surface Plasmon Resonance (SPR) measures changes in the refractive index of a metal surface (usually gold) caused by molecular mass binding [6,16,36,42,48]. SPR sensors provide label-free and real-time detection [6,16]. Localized SPR (LSPR) uses noble metal nanostructures (e.g., gold nanorods) [36]. Aptamers targeting PfLDH (e.g., 2008s) have been successfully integrated into SPR systems [37,48]. A dual-transducer aptasensor combined EIS (sensitive to 1 pM–100 nM) and surface plasmon polariton (SPP) detection (sensitive to 10 nM–1 M) on gold nanohole arrays for PfLDH detection, extending the overall dynamic range to six orders of magnitude [48].

Fluorescence and FRET (Förster Resonance Energy Transfer) rely on changes in light emission. In fluorescence-based aptasensors, binding causes an alteration (enhancement or quenching) in the fluorescent signal, often utilizing fluorescent dyes or carbon dots (CDs) conjugated to the aptamer [6,38,42]. FRET involves the non-radiative transfer of energy between a donor and acceptor fluorophore pair when they are in close proximity (typically <10 nm) [32,36]. This approach is leveraged in aptamer-based detection systems to signal target binding through conformational changes that alter the distance between the fluorophore and a quencher/acceptor [38,119]. This method has been used with *Plasmodium falciparum* lactate dehydrogenase (PfLDH), parasite nucleic acids, or other parasite-specific proteins.

Surface-Enhanced Raman Scattering (SERS) amplifies the intrinsically weak Raman signal generated by molecular vibrations when molecules are placed near nanostructured metal surfaces [6,16,36,42]. When combined with aptamers, SERS provides high sensitivity and label-free detection, particularly for hemozoin, demonstrating potential for ultrasensitive malaria diagnosis at parasite levels as low as 2.5 parasites/µL [6,13,16]. For SERS, the malaria application is much clearer because it has been directly used for hemozoin detection, a malaria-specific biomarker produced by *Plasmodium* parasites during hemoglobin digestion.

## 6. Characteristics of Aptamer-Based Biosensors (Aptasensors)

The following summarizes the methodologies and biosensing architectures used in aptamer-based diagnostic systems, with particular emphasis on immobilization strategies, signal amplification approaches, and assay design. It also discusses the principles that govern specificity and selectivity in aptasensing, including SELEX optimization, counter-selection strategies, and the integration of DNA nanostructures and amplification systems. These advances have significantly improved the sensitivity, selectivity, and portability of aptamer-based platforms, supporting their development as efficient alternatives for rapid and point-of-care diagnostics [25,26,27,28].

The shift toward aptamer-based diagnostics demands refined methodologies for immobilization, transduction, signal amplification, and validation, ensuring high sensitivity and specificity.

**Figure 5:**
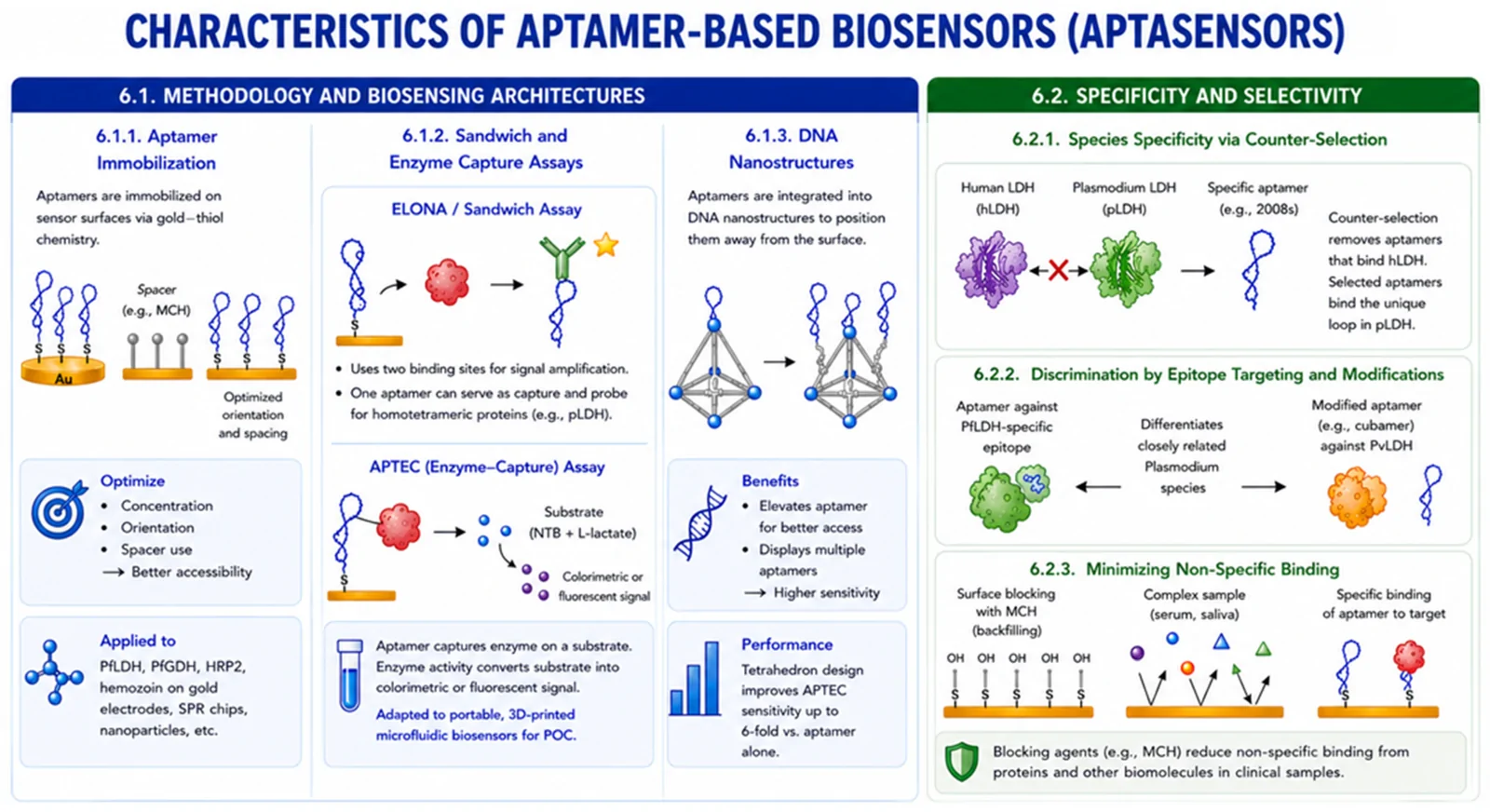
Characteristics of aptamer-based biosensors (aptasensors). The figure summarizes the main methodologies and biosensing architectures used in aptamer-based diagnostic platforms, including aptamer immobilization, sandwich and enzyme-capture assays, and DNA nanostructures for signal enhancement. It also highlights the principles governing specificity and selectivity, such as SELEX counter-selection, epitope targeting, chemically modified aptamers, and strategies to minimize non-specific binding. Together, these approaches improve the sensitivity, selectivity, portability, and applicability of aptasensors for rapid point-of-care diagnostics. The illustration was created using generative AI tools.

The characteristics of aptamer-based biosensors are summarized in Figure 5.

### 6.1. Methodology and Biosensing Architectures

A key feature of aptasensors is their integration into diverse formats beyond the standard laboratory ELISA (Enzyme-Linked Immunosorbent Assay), which is replaced by the analogous Enzyme-Linked Oligonucleotide Assay (ELONA) for screening binding affinities.

#### 6.1.1. Aptamer Immobilization

For sensing platforms, aptamers are immobilized onto the transducer surface, commonly via gold–thiol chemistry (covalent binding of thiolated aptamers to gold electrodes). To optimize performance, the aptamer concentration, orientation, and the use of spacers (e.g., mercaptohexanol (MCH)) must be carefully tailored to minimize steric hindrance and maximize binding site accessibility. In malaria biosensors, aptamers against biomarkers such as PfLDH, PfGDH, HRP2, or hemozoin are immobilized on gold electrodes, SPR chips, nanoparticles, or other sensing surfaces.

#### 6.1.2. Sandwich and Enzyme Capture Assays

ELISA/Sandwich Assays: Exploit two binding sites on the target for signal amplification. The standard ELISA sandwich format (antibody–antigen) is modified to incorporate aptamers, such as the aptamer–analyte–antibody hybrid system. For homotetrameric proteins like pLDH, a single aptamer sequence (e.g., 2008s) can serve both as capture and probe due to the protein’s repeating structure, simplifying the assay.

The APTEC (Aptamer-Tethered Enzyme Capture) Assay is a sensitive method where the aptamer captures the target enzyme (e.g., pLDH or PvLDH) onto a substrate. The intrinsic catalytic activity of the captured enzyme then converts an external substrate (e.g., nitrotetrazolium blue chloride (NTB) and L-lactate) into a colorimetric or fluorescent signal. The APTEC assay has been successfully adapted into portable, 3D-printed microfluidic biosensors for POC diagnosis [13,25,35].

#### 6.1.3. DNA Nanostructures

Aptamers are readily integrated into DNA nanostructures (e.g., tetrahedra, DNA origami) to spatially arrange the aptamer on the sensor surface. This approach can improve sensitivity by elevating the aptamer away from the surface to increase accessibility (e.g., for APTEC, an aptamer-based assay developed for the detection of *Plasmodium falciparum* lactate dehydrogenase (PfLDH)) or by displaying multiple aptamers [44,120]. The simplest design, a tetrahedron, was shown to improve APTEC sensitivity by up to 6-fold relative to the aptamer alone [120].

### 6.2. Structural and Molecular Selectivity

The usefulness of aptasensors for clinical malaria detection depends largely on their ability to distinguish between *Plasmodium* species and human proteins. For example, the 2008 aptamer was selected to specifically recognize a five-residue loop insertion in *Plasmodium* LDH that is absent in human LDH [46,47,117]. Targeting such parasite-specific structural features helps overcome the high sequence similarity between host and parasite proteins and can improve diagnostic accuracy, even in complex samples such as serum [46].

Generating aptamers against small, highly specific peptide epitopes (e.g., PfLDH-specific epitope) or utilizing chemically modified nucleotides (cubamers for PvLDH) enhances the ability to differentiate between closely related *Plasmodium* species (PfLDH vs. PvLDH) [24,47].

Employing techniques like backfilling the sensor surface with blocking agents (e.g., mercaptohexanol (MCH)) minimizes non-specific binding, which is particularly challenging in complex samples like serum or saliva due to the high background concentration of non-target proteins [4,43,117].

This high degree of specificity is critical for differential diagnosis—separating *P. falciparum* from non-*falciparum* infections—which guides appropriate treatment and management strategies [24]. In malaria biosensors, minimizing non-specific adsorption is essential because clinical samples (blood, serum, plasma, saliva) contain large amounts of proteins and other biomolecules that can interfere with the detection of malaria biomarkers such as PfLDH, PfGDH, HRP2, parasite DNA, or hemozoin.

### 6.3. Quantitative Correlation Between Biomarker Concentration and Parasite Density

Converting biomarker concentrations into parasite density is not a straightforward unit conversion but requires biomarker-specific biological assumptions. The estimated LOD in parasites/µL depends on the number of biomarker molecules produced per parasite, which varies across biomarkers and experimental conditions. Therefore, transparent reporting of the biological conversion factor and its source is essential to ensure reproducibility and enable meaningful comparisons between biosensing platforms.

In principle, a quantitative correlation can be established through experimental calibration by measuring the target protein concentration in samples with independently determined parasite densities (e.g., by microscopy or quantitative PCR). Statistical regression models can then be used to generate calibration curves relating biomarker concentration to parasite burden within defined clinical settings. Such approaches have been reported for malaria biomarkers including HRP2 and pLDH, although the relationship is not always strictly linear, particularly at low parasite densities or after treatment due to differences in biomarker persistence [121].

Establishing a robust correlation between biosensing signals and parasite counts is essential for clinical utility. For example, hemozoin accumulation is directly proportional to the maturation and cumulative development of the parasite population, allowing for a quantitative estimate of total parasite burden, even in cases of sequestration where parasites are absent from peripheral blood films. In contrast, for pLDH, the correlation is based on its role as a constitutive glycolytic enzyme produced by metabolically active parasites; because it is cleared within 24 h of treatment, its serum concentration provides a real-time snapshot of the active biomass.

## 7. Discussion: Critical Analysis of the Use of Aptamer-Based Biosensors for Detection of Malaria

This review adopts a biomarker-oriented perspective that connects the biological significance of *Plasmodium* targets with aptamer selection strategies and their implementation in different biosensing technologies. Emphasis is placed on the rationale for selecting diagnostic biomarkers, the relationship between target characteristics and aptamer performance, and the integration of aptamers into electrochemical, optical, magnetic, and microfluidic sensing platforms. Finally, emerging trends—including multiplexed detection, machine learning-assisted SELEX, nanomaterial-enabled aptasensors, and future point-of-care diagnostic strategies—are discussed to provide a forward-looking perspective on next-generation malaria diagnostics.

### 7.1. Biomarker Selection and Emerging Aptamer Targets for Aptamer-Based Malaria Diagnosis

The selection of an appropriate biomarker is one of the most critical factors determining the performance of aptamer-based malaria biosensors. An ideal target should be highly expressed during infection, absent from the human host, conserved among parasite isolates, and display a predictable relationship with parasite burden. Because no single biomarker fulfills all these requirements, recent research has focused on developing aptamers against multiple parasite-derived targets, each reflecting different aspects of parasite biology and therefore providing complementary diagnostic information [28,95].

Among the biomarkers investigated to date, *Plasmodium* lactate dehydrogenase (pLDH) remains the most extensively studied target. As a glycolytic enzyme produced exclusively by viable parasites, pLDH rapidly disappears following successful antimalarial treatment, making it particularly suitable for monitoring therapeutic response. Furthermore, the identification of species-specific epitopes has enabled the development of aptamers capable of distinguishing between *P. falciparum* and *P. vivax*, illustrating how rational epitope selection can improve analytical specificity while minimizing cross-reactivity with human lactate dehydrogenase [46,47,95]. Similar advantages have been reported for *Plasmodium falciparum* glutamate dehydrogenase (PfGDH), another metabolic enzyme expressed only during active infection, although considerably fewer aptamers have been developed against this biomarker [41].

In contrast, histidine-rich protein 2 (PfHRP2) remains the principal target of commercial rapid diagnostic tests because of its high abundance and excellent analytical sensitivity. However, unlike pLDH and PfGDH, PfHRP2 may persist in circulation for several weeks after parasite clearance, reducing its value for treatment monitoring and occasionally generating false-positive results. Moreover, the increasing prevalence of *pfhrp2* and *pfhrp3* gene deletions in several endemic regions has significantly compromised the reliability of HRP2-based diagnosis, reinforcing the need to identify alternative biomarkers capable of maintaining high diagnostic performance under these conditions [122].

Additional parasite-derived targets have also demonstrated considerable promise for aptamer development. PfHMGB1 exhibits limited sequence homology with its human counterpart and lacks several functionally important cysteine residues, providing opportunities for highly selective molecular recognition [50]. Likewise, the apicoplast enzyme DXR represents an attractive intracellular biomarker because of its parasite specificity and its essential role in isoprenoid biosynthesis, while hemozoin, the crystalline by-product of haemoglobin digestion, reflects cumulative parasite biomass and maturation rather than simple parasite viability. These biological differences suggest that combining biomarkers representing distinct metabolic pathways could substantially improve diagnostic robustness, particularly under conditions where individual targets show intrinsic limitations.

Because each biomarker exhibits different expression kinetics, persistence after treatment, and correlation with parasite density, quantitative biosensing requires careful calibration between biomarker concentration and parasitemia. Such relationships have already been established for PfHRP2 and pLDH using microscopy or quantitative PCR as reference methods, although they are not always linear because biomarker concentrations are influenced by parasite developmental stage, sequestration, antigen persistence, and host clearance mechanisms [80,81].

Table 6 summarizes the analytical performance of the principal malaria aptamers reported to date, including their affinity constants (K_d_), LODs, corresponding target biomarkers, and sensing platforms. Together, these data illustrate how biomarker selection directly influences analytical sensitivity, specificity, and clinical applicability.

Despite the remarkable progress achieved during the last decade, the range of parasite biomarkers explored for aptamer selection remains relatively limited. Future research should therefore expand to include novel parasite-specific targets, including aldolase, hemozoin-associated proteins, apicoplast enzymes, membrane-associated invasion proteins, and other intracellular proteins that remain largely unexplored. These biomarkers could provide complementary diagnostic information because they represent distinct biological processes involved in parasite metabolism, haemoglobin digestion, and erythrocyte invasion. Their incorporation into multiplexed biosensing platforms may improve diagnostic accuracy by simultaneously detecting multiple parasite biomarkers, thereby overcoming the limitations associated with individual targets such as *pfhrp2/pfhrp3* deletions or low-parasitemia infections.

Finally, although most current aptamer-based malaria biosensors focus on biomarkers expressed during the asexual blood stages, malaria elimination strategies also require the identification of asymptomatic transmission reservoirs. Mature gametocytes are responsible for parasite transmission to mosquitoes and frequently persist at low densities even after clinical symptoms have resolved [113]. To date, however, to the best of our knowledge, no aptamers have been reported against established gametocyte-specific biomarkers such as Pfs25 or Pfs230. The development of high-affinity aptamers targeting these transmission-stage molecules would represent an important advance, enabling multiplexed biosensors capable of simultaneously identifying parasite species, active infection, and transmission potential. Such platforms could transform aptamer-based diagnostics from tools for clinical case management into integrated systems supporting malaria surveillance and elimination programmes.

### 7.2. Analytical Performance and Technical Challenges of Aptamer-Based Biosensors

The excellent analytical performance reported for aptamer-based malaria biosensors demonstrates the remarkable potential of these molecular recognition elements for highly sensitive and selective parasite detection. Electrochemical, optical, surface plasmon resonance (SPR), surface-enhanced Raman scattering (SERS), field-effect transistor (FET), and mechanical transducers have all been successfully coupled with aptamers, achieving limits of detection ranging from the nanomolar to the femtomolar level, depending on the target biomarker and sensing platform [28,41,95]. The versatility of aptamers, together with their ease of chemical synthesis and functionalization, has facilitated their integration into increasingly sophisticated biosensing systems incorporating nanomaterials, DNA nanostructures, CRISPR/Cas amplification, Lab-on-a-Chip devices, and smartphone-assisted signal acquisition, thereby improving analytical sensitivity, automation, and portability [113,114,115,122].

Despite these advances, the successful translation of aptamer-based biosensors from controlled laboratory conditions to routine clinical practice remains challenging because of the complexity of biological matrices. Whole blood contains high concentrations of non-target proteins, including human serum albumin, together with red and white blood cells, electrolytes, and other biomolecules that can interfere with molecular recognition. The three-dimensional conformation of aptamers, which is essential for high-affinity target binding, is particularly sensitive to variations in pH, ionic strength, and buffer composition. Changes in these physicochemical parameters may alter aptamer folding, modify electrostatic interactions with the target, and reduce binding affinity, ultimately compromising sensor sensitivity [50,95].

Matrix effects are particularly relevant in electrochemical biosensors. High ionic strength shortens the Debye screening length, thereby reducing the effective electrical signal generated during target recognition in potentiometric devices such as aptamer-based field-effect transistors (aptaFETs). In addition, the high viscosity of whole blood and the presence of blood cells may interfere with analyte diffusion, generate hydrodynamic noise in acoustic devices, mask fluorescence signals, or physically obstruct immobilized recognition elements, reducing analytical performance. Consequently, biosensor evaluation using complex biological samples rather than buffered solutions is essential to assess their true clinical applicability.

Another important limitation is nonspecific adsorption, commonly referred to as biofouling. Proteins and other biomolecules present in blood may adsorb onto the sensor surface, generating background noise, signal drift, and reduced reproducibility. Although nucleic acid-based sensing surfaces generally exhibit lower nonspecific adsorption than antibody-based systems, the abundance of serum proteins and host enzymes remains a major challenge for clinical translation. Considerable efforts have therefore focused on optimizing surface chemistry to minimize these effects. Gold-thiol self-assembled monolayers combined with mercaptohexanol (MCH) effectively block unoccupied gold surfaces, improve aptamer orientation, and reduce nonspecific protein adsorption. Likewise, DNA nanostructures, particularly tetrahedral DNA scaffolds, elevate the aptamer away from the electrode surface, improving target accessibility while simultaneously reducing steric hindrance and biofouling [28,95].

Additional improvements have been achieved through the incorporation of microfluidic sample-processing systems. These devices enable the concentration of infected erythrocytes, selective extraction of intracellular parasite biomarkers, and controlled manipulation of biological fluids before detection, thereby reducing matrix interference while simplifying sample preparation. Such integrated platforms are particularly attractive for point-of-care applications because they minimize operator intervention and improve assay reproducibility.

Overall, sensor performance depends not only on aptamer affinity but also on immobilization chemistry, transducer design, sample preparation, and resistance to matrix-induced interference. The principal technical challenges affecting aptamer-based malaria biosensors include aptamer conformational stability, nonspecific adsorption, biofouling, and sample complexity, together with the engineering strategies currently employed to overcome these limitations. Continued advances in surface chemistry, nanostructured materials, microfluidics, and signal amplification technologies are expected to further improve the robustness, reproducibility, and clinical applicability of aptamer-based malaria diagnostics.

### 7.3. Clinical Translation, Field Implementation, and Future Perspectives

Despite the considerable advances achieved in aptamer discovery and biosensor engineering, the translation of aptamer-based malaria biosensors from proof-of-concept studies to routine clinical practice remains limited. To date, to the best of our knowledge, no aptamer-based biosensor has been approved for clinical malaria diagnosis, and most reported platforms have been evaluated under controlled laboratory conditions using spiked samples or relatively small cohorts of clinical specimens [28,95,122]. Consequently, large-scale clinical validation under real-world conditions remains an essential step before these technologies can be incorporated into routine diagnostic workflows.

Aptamers demonstrate significant quantitative advantages in stability over traditional antibody-based reagents, addressing the primary environmental stressors of malaria-endemic regions. While conventional antibody-based RDTs typically possess a restricted shelf life of approximately nine months, DNA aptamers have been documented to remain functional at room temperature for several years [45]. Specifically, DNA-based biosensor platforms, such as those utilizing a quartz crystal microbalance to target the *Plasmodium falciparum msp2* gene, have confirmed storage stability of at least 180 days [3,13,58]. Furthermore, aptamers exhibit remarkable thermal resilience, uniquely characterized by their ability to undergo reversible folding; they can recover their native, functional three-dimensional conformation even after being subjected to extreme denaturation at 95 °C [2]. In contrast, monoclonal antibodies suffer from irreversible denaturation and permanent loss of binding affinity when exposed to the high heat and humidity typical of tropical climates. Despite these inherent biochemical advantages, it must be explicitly noted that most stability assessments to date have been conducted under controlled laboratory conditions or in simplified buffer systems. There is currently a lack of comprehensive longitudinal field data documenting the performance and sensitivity of these aptasensors over their entire intended lifetime while they are exposed to fluctuating real-world environmental stresses. Consequently, evaluating long-term stability within complex biological matrices and actual field environments remains a critical knowledge gap that must be addressed to ensure these sensors meet the World Health Organization’s ASSURED criteria for clinical impact.

Although aptamers possess several intrinsic advantages over antibodies, including chemical synthesis, low batch-to-batch variability, excellent thermal stability, ease of chemical modification, and reversible folding following thermal denaturation, the performance of an aptasensor depends on much more than the recognition element alone. Immobilization chemistry, transducer stability, device packaging, storage conditions, and long-term analytical reproducibility all contribute to the overall performance of the sensing platform. Importantly, most available stability studies have been conducted under controlled laboratory conditions, whereas long-term storage and operation under the elevated temperatures, high humidity, and limited infrastructure characteristic of malaria-endemic regions remain insufficiently investigated.

The implementation of aptamer-based biosensors in field settings also requires overcoming several operational challenges. Many high-performance sensing platforms continue to rely on sophisticated instrumentation, specialized readers, laboratory-based sample preparation, or highly trained personnel, increasing both cost and operational complexity compared with conventional rapid diagnostic tests (RDTs).

Another important consideration is the validation of biosensors using clinically relevant biological matrices, which include a high concentration of non-target proteins (such as human serum albumin), cells (red and white blood cells), and varying ionic environments. These components can profoundly influence aptamer folding, facilitate nonspecific adsorption, and cause sensor fouling, often resulting in decreased sensitivity and increased limits of detection (LOD) compared to controlled laboratory buffers.

Variations in pH and ionic strength can alter aptamer folding, target protein charge, and electrostatic interactions, thereby affecting binding affinity and signal transduction. In addition, nonspecific adsorption (biofouling) by abundant serum proteins can reduce sensitivity and specificity, although surface blocking agents (e.g., MCH) and DNA nanostructures help mitigate these effects. Finally, the viscosity and cellular components of whole blood, particularly red blood cells, can interfere with signal detection, making sample pre-treatment and microfluidic separation strategies valuable for improving analytical performance.

As discussed in the previous section, matrix-induced interference, operator variability, and environmental conditions may substantially influence analytical performance. Consequently, future studies should extend beyond analytical sensitivity and specificity measured under laboratory conditions to include robustness, reproducibility, ease of use, manufacturing scalability, regulatory approval, and cost-effectiveness. Addressing these aspects will be essential for translating promising laboratory prototypes into reliable diagnostic tools suitable for widespread deployment in endemic regions.

The transition of laboratory-validated biosensors to clinical use is further complicated by practical environmental and human constraints in endemic settings. Field studies conducted with village malaria workers have demonstrated that current device packaging, often made of paper, is easily destroyed by rain and humidity, necessitating a transition toward more durable plastic casing for field use. Operational failures frequently arise from human–machine interface issues, such as the difficulty health workers face in handling small pipettes, making precise buffer measurements, or identifying a lack of battery status notifications, all of which can lead to erroneous diagnostic readings in remote areas. In addition, sample collection, buffer preparation, reagent stability, battery management, calibration procedures, and environmental durability may significantly affect analytical performance under point-of-care conditions. Although aptamers themselves are relatively inexpensive recognition elements, the affordability and accessibility of the complete diagnostic platform ultimately depend on successful integration of all device components. A significant “cost-sensitivity paradox” persists in the diagnostics market, where the high upfront investment required for specialized readers—such as Lab-on-Chip (LoC), portable PCR, or magnetic resonance systems—clashes with the limited resources of healthcare providers in regions where consumers are often only willing to pay an average of $0.53 per test [16]. Furthermore, the complexity of biological matrices like whole blood or saliva often degrades sensor performance compared to controlled laboratory buffers. In potentiometric systems such as aptaFETs, the high ionic strength of human serum can lead to a shortened Debye screening length, which effectively shields the captured protein’s charge and significantly reduces analytical sensitivity [6].

A prioritized research roadmap must address these barriers through standardized sample pre-treatment and the strategic selection of species-specific targets. Integrating automated, on-chip preparation modules with isothermal amplification techniques like LAMP—which are notably more robust to typical blood inhibitors than traditional PCR—represents a vital step toward portable, electronic “sample-to-result” diagnostics [34,123]. Future aptamer selection methodologies should shift away from whole-protein targets toward conserved, species-specific peptide epitopes, such as the unique LISDAELEAIFDC loop in PfLDH, to ensure reliable differentiation between *P. falciparum* and *P. vivax*, which is necessary for administering radical cure treatments to eliminate liver-stage hypnozoites [25].

The future of malaria surveillance lies in the convergence of non-invasive multiplexing and digital health integration. Developing sensors for saliva or urine that can simultaneously detect parasite biomarkers and drug-resistance genes, such as the PfK13 marker, would revolutionize active screening by eliminating the biohazards and cultural reluctance associated with blood collection [112]. Finally, leveraging machine learning to optimize the binding interfaces of aptamers and deploying smartphone-integrated readers for real-time, cloud-based reporting will be essential for the World Health Organization’s global 2030 elimination targets [34].

Future developments should therefore focus on the design of robust, fully integrated point-of-care platforms combining automated microfluidic sample preparation, portable electrochemical or optical transducers, miniaturized electronics, smartphone-assisted data analysis, and cloud-based reporting systems. Advances in machine learning-assisted SELEX are expected to accelerate the identification of high-affinity, highly selective aptamers while facilitating the rational design of aptamers against novel parasite-specific epitopes. At the same time, multiplexed biosensors capable of simultaneously detecting conserved metabolic enzymes, species-specific biomarkers, and transmission-stage antigens may overcome current diagnostic limitations associated with *pfhrp2/pfhrp3* deletions, low-parasitemia infections, and asymptomatic parasite carriage. The integration of these technological advances has the potential to transform malaria diagnostics from simple case detection into comprehensive platforms supporting surveillance, treatment monitoring, transmission assessment, and ultimately malaria elimination.

## 8. Conclusions

Malaria, predominantly caused by *Plasmodium falciparum*, remains a major global health challenge, accounting for substantial morbidity and mortality worldwide. Effective malaria control and eradication efforts depend critically on the availability of prompt, sensitive, and specific diagnostic tools capable of detecting both symptomatic and asymptomatic infections. However, conventional diagnostic approaches—namely light microscopy (LM) and rapid diagnostic tests (RDTs)—are frequently limited by insufficient sensitivity, particularly in cases of low-level parasitemia, and by vulnerabilities such as antigenic variability, including deletions of the *pfhrp2* gene. These shortcomings have driven the rapid evolution of the malaria diagnostics landscape and intensified the search for more robust and ultrasensitive alternatives.

In this context, advanced diagnostic platforms based on biosensor technologies have emerged as promising solutions, offering high sensitivity, rapid analysis, portability, and suitability for point-of-care (POC) deployment. Central to these innovations is aptamer technology, which represents a paradigm shift in molecular recognition. Aptamers are single-stranded nucleic acids generated through the Systematic Evolution of Ligands by Exponential Enrichment (SELEX) process and are capable of binding targets with affinities and specificities comparable to those of antibodies. Importantly, aptamers offer distinct advantages, including high thermal and chemical stability, low batch-to-batch variability, reduced production costs, and ease of chemical modification, making them particularly attractive for integration into diagnostic platforms designed for resource-limited settings.

Recent advances have enabled the development of aptamers targeting a wide range of malaria biomarkers, encompassing metabolic enzymes such as parasite lactate dehydrogenase (pLDH), *Plasmodium falciparum* glutamate dehydrogenase (PfGDH), and 1-deoxy-D-xylulose 5-phosphate reductoisomerase (DXR), as well as structural and regulatory components including high mobility group box 1 (PfHMGB1) and the malaria pigment hemozoin.

Future developments in aptamer-based malaria diagnostics are expected to expand to include novel parasite-specific biomarkers that remain largely unexplored, including aldolase, hemozoin-associated proteins, apicoplast enzymes, and membrane-associated invasion proteins. The selection of high-affinity aptamers against these emerging targets could improve species discrimination, enhance detection sensitivity during low-parasitemia infections, and enable multiplexed biosensing platforms capable of simultaneously identifying multiple stages or species of *Plasmodium*. In particular, biomarkers such as aldolase and hemozoin may provide complementary diagnostic information to that obtained from PfHRP-2 or PfLDH because of their direct association with parasite metabolism and hemoglobin digestion pathways.

The incorporation of malaria-specific aptamers into electrochemical, optical, and mechanical biosensing platforms—such as field-effect transistor (FET)-based sensors, multiplexed surface plasmon resonance (SPR), and surface-enhanced Raman scattering (SERS)—has enabled quantitative and ultrasensitive parasite detection, often achieving picomolar to femtomolar sensitivity ranges.

Collectively, these developments underscore the transformative potential of aptamer-based biosensors as next-generation diagnostic tools. Continued research efforts focused on validating aptasensors with clinically relevant samples and integrating them into fully portable, robust POC devices will be essential to translate laboratory advances into real-world impact. Such progress is crucial for meeting the World Health Organization’s ambitious targets for malaria control and, ultimately, for supporting global malaria elimination programs.

## Figures and Tables

**Table 1:** Representative aptamers developed against *Plasmodium* biomarkers and parasite-associated targets for malaria diagnostics and biosensing applications.

Aptamer Class	Target Biomarker (Antigen)	Specific Aptamers	Binding Affinities/ Key Features	Source Citations
Enzyme/Protein (LDH)	*Plasmodium* Lactate Dehydrogenase (pLDH)	2008s (DNA), pL1 (DNA)	Pan-specific detection of *Plasmodium* species; 2008s (PfLDH) binding discriminates hLDH	[13,46]
Enzyme/Protein (LDH-Epitope)	PfLDH species- specific epitope (LISDAELEAIFDC)	LDHp 11 (DNA)	Species-specific targeting (PfLDH over PvLDH); to recombinant PfLDH	[24]
Enzyme/Protein (LDH-Modified)	*P. vivax* LDH (PvLDH)	Cubamer (1501s) (Cubane-modified DNA)	Discrimination between PvLDH and PfLDH, utilizing synthetic cubane units for novel binding (PvLDH)	[47]
Enzyme/Protein (GDH)	PfGDH	NG3 (ssDNA)	Highly selective, low fM LOD in electrochemical FET biosensors	[13]
DNA-Binding Protein	PfHMGB1 (HMG-box domain)	PfR6, PfE3 (DNA)	Potential novel biomarker, high expression, high affinity (range)	[52]
Apicoplast Enzyme	DXR (1-deoxy- D-xylulose-5- phosphate reductoisomerase)	D10 (DNA)	Targets DXR (essential MEP pathway enzyme); localizes to the apicoplast	[51]
Heme Pathway	Heme (Fe- protoporphyrin IX)	PS2R, PS2M, PS21, PS26, OKA26-3, OKA-26-5 (DNA)	Binds heme, inhibits hemozoin formation, reducing parasite viability	[12]
Whole Cell Surface	Infected Erythrocyte (IE) Surface Ligands (e.g., PfEMP1, var2CSA)	Malaria.1, 8.1-1 19, 24, 30, 77, and 78 (RNA/DNA)	Broad binding across lab strains/clinical isolates; targets surface proteins like var2CSA (placental sequestration protein)	[49,50]
Metabolite/Lipid	Linear Polyisoprenoids (Dolichols/ Polyprenols)	AptPP (DNA)	Targets isoprenoid end-products, useful for subcellular localization/imaging, high selectivity	[53]

**Table 3:** Comparative features of *Plasmodium* (PfGDH) and human glutamate dehydrogenases (GDH).

Feature	*Plasmodium* *falciparum* GDH (PfGDH)	Human GDH (hGDH1/hGDH2, etc.)	Implications (Diagnostics/ Inhibitors/Biosensors)
Cofactor specificity	Strictly NADP- dependent (NADP^+^/NADPH) in major PfGDH isoforms [72].	Dual specificity (can use NAD^+^ or NADP^+^) depending on isoform and tissue context [73].	The NADP dependence of PfGDH allows design of assays or inhibitors that avoid interfering with host enzymes using NAD^+^.
Hexameric oligomerization & subunit interfaces	PfGDH has salt bridge-rich interfaces, a unique N-terminal extension, and two active-site differences [74].	GDH has mainly hydrophobic interfaces, no N-terminal extension, and additional regulatory elements such as the antenna [75].	Unique structural features provide epitopes or binding surfaces for selective inhibitors or aptamers that do not bind human GDH.
Kinetics	PfGDH2 has $K_{m}$ somewhat higher than PfGDH1; enzyme is efficient; specific activity comparable between isoforms [72].	Human isoforms differ: hGDH1 and hGDH2 have $\mathrm{different}\;K_{m}$ for glutamate, α-ketoglutarate, and ammonium; also vary with allosteric effectors [76].	Differences in enzyme kinetics influence biosensor performance and should be considered when designing selective inhibitors.
Regulation/ allostery	PfGDH shows limited allosteric regulation and is likely regulated primarily by substrate availability and metabolic state rather than by classical effectors such as GTP and ADP [74].	Highly regulated by allosteric effectors: ADP (activator), GTP (inhibitor), leucine; conformational shifts upon binding effectors; various isoform and tissue regulations [75].	In targeting PfGDH, inhibitors or aptamers can exploit differences in regulation; avoiding binding to human GDH regulatory sites helps reduce toxicity.
Localization/role	PfGDH isoforms are located in different compartments: cytosol and apicoplast; important source of NADPH for antioxidative stress defence; supports parasite metabolic needs [72].	GDH is a mitochondrial enzyme involved in glutamate and ammonia metabolism, linking amino acid metabolism with central carbon metabolism, neurotransmitter synthesis, and the urea cycle [73].	Selectivity in diagnostics or drug design can exploit parasite localization (e.g., apicoplast vs. mitochondrial), or dependency of parasite on PfGDH for redox homeostasis.

**Table 6:** Comparative analytical performance of representative aptamer-based biosensors for malaria detection. A comprehensive description of the properties of aptamer-based biosensors, including sensitivity, sample complexity, and device design, was recently reported [122]. Abbreviations: SPR: surface plasmon resonance; EIS: Electrochemical Impedance Spectroscopy; FET: Field-Effect Transistor; ITC: Isothermal Titration Calorimetry; EMSA: Electrophoretic Mobility Shift Assay; N/R: not reported.

Target Biomarker	Aptamer	Kd	Sensing Platform	LOD	Linear Range	Reference
PfGDH	NG3 (DNA)	79.2 ± 1.6 nM (SPR)	EIS/FET	100 fM	100 fM–100 nM	[13,44]
PfLDH	2008s (DNA)	42 nM (ITC); 53 ± 3 nM (EMSA); 59 nM (SPR)	SPR/EIS	1 pM	1 pM–1 μM	[13,38]
pLDH	LDHp11 (DNA)	321 ± 83 nM (PfLDH); 37 ± 41 nM (PvLDH)	Optical/Paper-based	Low pM	Species-specific	[23,49,50]
PfHRP2	B4	1.32 μM	Electrochemical	Picomolar	N/R	[72,73,74]
	2106s	29.53 nM (SPR)	Electrochemical	Picomolar	N/R	[72,73,74]
PfHMGB1	PfR6	N/R	Electrochemical/SPR/Fluorescence	nM–pM	N/R	[52,88]
	PfE3	N/R	Electrochemical/SPR/Fluorescence	nM–pM	N/R	[52,88]
DXR	D10	N/R	Fluorescence imaging	Qualitative	N/R	[51]

## Data Availability

No new data were created or analyzed in this study. Data sharing is not applicable to this article

## Abbreviations

The following abbreviations are used in this manuscript:

APTEC	Aptamer-Tethered Enzyme Capture
ASSURED	Affordable, Sensitive, Specific, User-friendly, Rapid and Robust, Equipment-free, and Deliverable
ATP	Adenosine Triphosphate
AuNP	Gold Nanoparticle
CMOS	Complementary Metal-Oxide Semiconductor
CDs	Carbon Dots
DAMP	Damage-Associated Molecular Pattern
DNA	Deoxyribonucleic acid
DXP	1-deoxy-D-xylulose-5-phosphate
DXR	1-deoxy-D-xylulose-5-phosphate reductoisomerase
DXS	1-deoxy-D-xylulose-5-phosphate synthase
EC	Enzyme Commission
EgFET	Extended Gate Field-Effect Transistor
EIS	Electrochemical Impedance Spectroscopy
ELISA	Enzyme-Linked Immunosorbent Assay
ELONA	Enzyme-Linked Oligonucleotide Assay
EMSA	Electrophoretic Mobility Shift Assay
FAM	Fluorescein Amidite
FET	Field-Effect Transistor
FNR	Ferredoxin-NADP Reductase
FRET	Förster Resonance Energy Transfer
G3P	Glyceraldehyde-3-phosphate
GDH	Glutamate dehydrogenase
GTP	Guanosine Triphosphate
HDL	High-density lipoprotein
HMGB1	High mobility group box 1 protein
hGDH	Human Glutamate Dehydrogenase
hLDH	Human Lactate Dehydrogenase
HRP2	Histidine-Rich Protein 2
IEs	Infected Erythrocytes
IPP	Isopentenyl Pyrophosphate
ISFET	Ion-Sensitive Field-Effect Transistor
I-SELEX	Inertial Microfluidic SELEX
ITC	Isothermal Titration Calorimetry
kDa	Kilodalton
Km	Michaelis constant
LAMP	Loop-mediated isothermal amplification
LDH	Lactate dehydrogenase
LDL	Low-density lipoprotein
LM	Light microscopy
LLOD	Lower Limit of Detection
LOD	Limit of Detection
LSPR	Localized Surface Plasmon Resonance
MCH	Mercaptohexanol
MEP	Methylerythritol Phosphate
MRR	Magnetic Resonance Relaxometry
NAD+	Nicotinamide Adenine Dinucleotide
NADH	Reduced Nicotinamide Adenine Dinucleotide
NADP+	Nicotinamide Adenine Dinucleotide Phosphate
NADPH	Reduced Nicotinamide Adenine Dinucleotide Phosphate
NG3	Name of an aptamer specific against PfGDH
NTB	Nitro Tetrazolium Blue chloride
PCR	Polymerase Chain Reaction
PDB	Protein Data Bank
Pf	*Plasmodium falciparum*
PfGDH	*Plasmodium falciparum* Glutamate Dehydrogenase
PfHMGB1	*Plasmodium falciparum* High mobility group box 1 protein
PfHRP2	*Plasmodium falciparum* Histidine-Rich Protein 2
PfLDH	*Plasmodium falciparum* Lactate Dehydrogenase
pLDH	*Plasmodium* Lactate Dehydrogenase
POC	Point of Care
pRBC	Parasitized Red Blood Cell
PvLDH	*Plasmodium vivax* Lactate Dehydrogenase
QCM	Quartz Crystal Microbalance
RBC	Red Blood Cell
RCA	Rolling Circle Amplification
RDTs	Rapid Diagnostic Tests
REEAD	Rolling Circle-Enhanced Enzyme Activity Detection
RNA	Ribonucleic Acid
RT-PCR	Reverse Transcription Polymerase Chain Reaction
SELEX	Systematic Evolution of Ligands by Exponential Enrichment
SERS	Surface-Enhanced Raman Scattering
SPR	Surface Plasmon Resonance
SPP	Surface Plasmon Polariton
ssDNA	Single-Stranded DNA
TAAG	Triacylglyceride
TLR4	Toll-Like Receptor 4
TNF-α	Tumor Necrosis Factor alpha
WHO	World Health Organization
